# Revealing the global mechanism related to carnosine synthesis in the pectoralis major of slow-growing Korat chickens using a proteomic approach

**DOI:** 10.5713/ab.24.0119

**Published:** 2024-08-14

**Authors:** Panpradub Sinpru, Chanadda Suwanvichanee, Rujjira Bunnom, Satoshi Kubota, Jirawat Yongsawatdigul, Wittawat Molee, Kanjana Thumanu, Amonrat Molee

**Affiliations:** 1 School of Animal Technology and Innovation, Institute of Agricultural Technology, Suranaree University of Technology, Nakhon Ratchasima, 30000, Thailand; 2 School of Food Technology, Institute of Agricultural Technology, Suranaree University of Technology, Nakhon Ratchasima, 30000, Thailand; 3 Synchrotron Light Research Institute (Public Organization), Nakhon Ratchasima, 30000, Thailand

**Keywords:** Carnosine Content, Molecular Function, Molecular Pathway, Proteome, Slow-growing Chicken

## Abstract

**Objective:**

This study aimed to find global mechanisms related to carnosine synthesis in slow-growing Korat chickens (KRC) using a proteomic approach.

**Methods:**

*M. pectoralis major* samples were collected from 10-week-old female KRC including low-carnosine (LC, 2,756.6±82.88 μg/g; n = 5) and high-carnosine (HC, 4,212.5 ±82.88 μg/g; n = 5).

**Results:**

We identified 152 common proteins, and 8 of these proteins showed differential expression between the LC and HC groups (p<0.05). Heat shock 70 kDa protein 8, Heat shock 70 kDa protein 2, protein disulfide isomerase family A, member 6, and endoplasmic reticulum resident protein 29 were significantly involved in protein processing in the endoplasmic reticulum pathway (false discovery rate<0.05), suggesting that the pathway is related to differential carnosine concentration in the *M. pectoralis major* of KRC. A high concentration of carnosine in the meat is mainly involved in low abundances of Titin isoform Ch12 and Connectin and high abundances of M-protein to maintain homeostasis during muscle contraction. These consequences improve meat characteristics, which were confirmed by the principal component analysis.

**Conclusion:**

Carnosine synthesis may occur when muscle cells need to recover homeostasis after being interfered with carnosine synthesis precursors, leading to improved muscle function. To the best of our knowledge, this is the first study to describe in detail the global molecular mechanisms in divergent carnosine contents in meat based on the proteomic approach.

## INTRODUCTION

Slow-growing Korat chicken (KRC) is a crossbred chicken between the Thai indigenous sire and a dam from the synthetic breed developed by Suranaree University of Technology. KRC meat contains less fat, low purine, and higher quantities of protein than commercial broilers since it is predominantly α-helices [1]. Developing nutrient-rich chicken meat, which is fast-growing in the healthy-food industry, has increased the competition with other healthy products [2]. Therefore, improving nutrient-rich KRC meat is a good strategy to promote a diet with healthy attributes together with increasing the competitiveness of slow-growing chicken against commercial chicken breeds in Thailand.

Carnosine is a naturally occurring dipeptide histidine present in the body and is syn thesized from a combination of β-alanine and L-histidine using the enzyme carnosine synthase [3]. Carnosine is only available in foods derived from animal products, which are mainly found in high concentrations in skeletal muscles [4]. Carnosine is beneficial for human health as it can suspend biochemical processes via scavenging free radicals, chelating metals, and maintaining buffer capacity [5]. A previous study has reported that genetics is one of the sources of variation in carnosine content in animals [6]. The molecular processes involved in carnosine synthesis in fast-growing chickens were described by Cong et al [7], Qi et al [8], and Qi et al [9]. They found that carnosine synthesis was related to the expression of the genes encoding histidine decarboxylase, proton-coupled oligopeptide transporters, solute carrier family 6 member 6, and carnosine synthase. Additionally, Khumpeerawat et al [10] and Sharma et al [11] found that the genes carnosine synthase1 and solute carrier family 36, member 1 transporter were involved in carnosine synthesis in slow-growing chickens. Moreover, Drozak et al [12] reported that the ATP-grasp domain-containing protein 1 (ATPGD1) was related to carnosine synthesis. All the above-mentioned genes and proteins are specific to carnosine synthesis; however, the holistic pathways, genes or proteins involved in carnosine synthesis, carnosine function, and its consequences still need to be understood.

In KRC, carnosine synthesis precursors (β-alanine and 0.5% L-histidine) in diet can be absorbed and transported through the small intestine to elevate the level of carnosine in breast meat [13]. A study by our group reported that an increase in carnosine content in meat can decrease pH (pH_45 min_) and the value of thiobarbituric acid reactive substances (TBARS) that consequently prevents a change in the protein secondary structure, resulting in improved water-holding ability in the breast meat of KRC [14]. These results align with those obtained by Cong et al [7] and Qi et al [8], which suggest that an increase in carnosine content in the *M. pectoralis major* muscle of chicken plays a role in regulating post-mortem pH decline that induces muscle metabolism during the pre-rigor phase and consequently improves meat characteristics. Moreover, transcriptomics or proteomics is a powerful approach to reveal global pathways. To date, only one study by Kubota et al [15] has described the transcriptomic profile related to carnosine synthesis in the breast meat of female KRC fed with L-histidine or β-alanine as dietary supplements. Pathways associated with meat tenderness and oxidative stress resistance were discovered but that of carnosine synthesis were not found. However, Suwanvichanee et al [14] determined that supplementing with the combination of β-alanine and L-histidine produced the highest carnosine content in KRC meat, and both amino acids were also considered limiting factors for carnosine synthesis in chickens [16]. As proteins and enzymatic biological functions mostly determine the phenotypic diversity from a set of common genes [17], we hypothesized that protein changes and their function related to physiological and biological processes may contribute to differential carnosine content in KRC breast meat fed with L-histidine or β-alanine.

The current study aimed to find the global pathways related to carnosine synthesis, molecular functions, and their consequences on muscle structure and functions in *M. pectoralis major* of slow-growing KRC using a proteomic approach. To the best of our knowledge, we described for the first-time global protein expressions and muscle functions with different carnosine contents in meat. This work lays a theoretical foundation for the genetic improvement of carnosine content in slow-growing chickens.

## MATERIALS AND METHODS

### Ethics approval

The experiments and animal care protocol were approved by the Ethics Committee on Animal Use of Suranaree University of Technology, Nakhon Ratchasima, Thailand; document ID: 18/2560. All methods in the study were performed in accordance with appropriate guidelines. This study is reported in accordance with ARRIVE guidelines (https://arriveguidelines.org).

### Animals and sample selection

Ten *M. pectoralis major* samples from female KRC were selected based on the significant difference in carnosine content from our previous study [14]. Briefly, female chickens in low-carnosine (LC) and high-carnosine (HC) groups were fed with a basal diet and a basal diet supplemented with 1.0% β-alanine (Sigma-Aldrich, St. Louis, MO, USA; 146064) + 0.5% L-histidine (AppliChem GmbH, Darmstadt, Germany; A3738), respectively. A detailed description of the formulation diets is shown in Supplementary Table S1. At 10 weeks of age, the chickens were slaughtered, and *M. pectoralis major* samples were collected for meat characteristics and carnosine content measurements. For this study, 10 female KRC breast meat samples were separated into 2 groups based on the carnosine content: LC = 2,756.6±82.88 μg/g (n = 5) and HC = 4,212.5±82.88 μg/g (n = 5). The data on meat characteristics and carnosine content was published by Suwanvichanee et al [14] as shown in Supplementary Table S2. For proteomic analysis, breast meat tissue of approximately 10 g from each sample was collected from similar parts of the breast used for the carnosine content measurement. They were then snap-frozen in liquid nitrogen and kept frozen at −80°C until used for protein extraction.

### Protein extraction

Proteins were extracted according to a previously described method [18]. Breast meat samples were freeze-dried using the freeze dryer GAMMA 2–16 LSCplus (Martin Christ, Gefriertrocknungsanlagen GmbH, Osterode, Germany) and finely ground to a powder. Then the breast powder was ice-sonicated in 50 mM ammonium bicarbonate buffer (AMBIC) containing 8 M urea (Sigma-Aldrich, Saint Louis, MO, USA). The extract proteins were centrifuged at 20,000×g for 10 min at 4°C. The supernatants were collected and diluted with 50 mM ammonium bicarbonate buffer to a final concentration of 1.5 M urea. Then, the concentrations of protein were measured using the Pierce BCA Protein Assay kit (Thermo Fisher Scientific, Waltham, MA, USA). From each supernatant sample, 100 μg of protein was reduced to disulfide bonds by adding dithiothreitol to a final concentration of 5 mM and incubated between 50°C and 60°C for 20 min. Then, iodoacetamide was added to a final concentration of 15 mM and was incubated in the dark at 25°C for 20 min. Subsequently, 2 μg of trypsin (Promega, Madison, WI, USA) was added and kept overnight at 37°C to digest the protein sample into peptides.

### Mass spectrometry

Each protein sample (4 μg) was desalted with C18 columns and separately analyzed with a TripleTOF 6600 Quadrupole Time-Of-Flight instrument (Sciex, Framingham, MA, USA) coupled to an Eksigent nanoLC (Eksigent Technologies, Dublin, CA, USA) with a Turbo V Mass spectrometry analysis performed in the microspray mode at the Proteomics Unit core facility, University of Helsinki, Finland. YMC-Triart C18 column (12 nm, 3 μm, 150×0.3 mm) (YMC CO, Kyoto, Japan) was used for peptide separation. The MS-data-independent acquisition (DIA) in positive ion mode, using a linear 60-min gradient from 5% to 35% buffer B (0.1% formic acid in acetonitrile). An accumulation time of 250 ms was used for survey scans acquired and the top 30 ions above the intensity threshold of 150 counts were selected for subsequent MS/MS scans (100 to 1,500 m/z, 100 ms accumulation time per MS/MS).

### Protein identification

The raw wiff files were obtained using MaxQuant software version 1.6.5.0 [19] for protein identification. Peptides were identified using UniProtKB *Gallus gallus* database (total 34,827 entries, downloaded in March 2022 from https://www.uniprot.org). The parameters of protein identification were set as trypsin specificity enzyme with 2 missed cleavages. Carbamidomethylation of cysteine residues and oxidation of methionine were used as static modification and dynamic modification, respectively. The first search and main search were set to 0.07 and 0.006 Da, respectively. The fragment (MS/MS) mass deviation was set to 20 ppm; peptide and protein false discovery rate (FDR) were both set at 1%. Match-between-runs algorithm was used with a match time window of 0.7 min and an alignment time search space of 20 min. Razor and unique peptide were used for label-free quantification (LFQ) algorithm (minimum ratio count = 2).

### Differential proteomic analyses

Statistical analysis and data visualization were performed in Perseus software version 1.6.5.0 (Tyanova et al [20]). LFQ intensity data generated from Maxquant were loaded into Perseus. The data was filtered by removing proteins identified with post-translation modification, contaminant proteins, or hits related to the reverse sequence. For quantification, proteins were kept if they appeared in at least 2 out of 5 biological replicates of both experimental groups. The data was transformed to a logarithmic scale with base 2. Missing values were then imputed. For the comparison between LC and HC groups, proteins with a fold change ≥1 and p<0.05 were considered as the statistical significance of differentially abundant proteins (DAPs). Hierarchical clustering was then conducted with DAPs after Z-score normalization.

### Bioinformatics analysis

The lists of DAPs and exclusive proteins of LC and HC groups were combined for functional enrichment analysis. The significant difference in the enriched gene ontology (GO) terms composed of biological process, cellular component, molecular function, and Kyoto encyclopedia of genes and genomes (KEGG) pathway was considered at p adjusted by FDR<0.05 [21]. The interactions between protein and protein networks (PPI) of DAPs and exclusive proteins in each group were identified using STRING version 10 (https://string-db.org) against the database of *Gallus gallus*, and the interactions were considered at a medium confidence score of 0.4 [22].

### Principal component analysis

The principal component analysis (PCA) was applied for clustering and investigating the relationship among parameters of carnosine content, meat characteristics (pH_45 min_, pH_24 h_, drip loss, cooking loss, and shear force), TBARS value, secondary structure ratio (α-helix, β-sheet, β-turn), and DAPs intensity using Unscrambler X Multivariate Data Analysis software (version 10.1; Camo Analytics, Oslo, Norway). The data used for PCA analysis as shown in Supplementary Table S3 were from our previous study by Suwanvichanee et al [14]. The related variables among the data were represented by a correlation loading plot. All the variable data were weighted using a standard deviation weighting process before the analysis using PCA bi-plot correlation.

## RESULTS

### Analysis of differential proteins involved in carnosine synthesis

**Figure 1:**
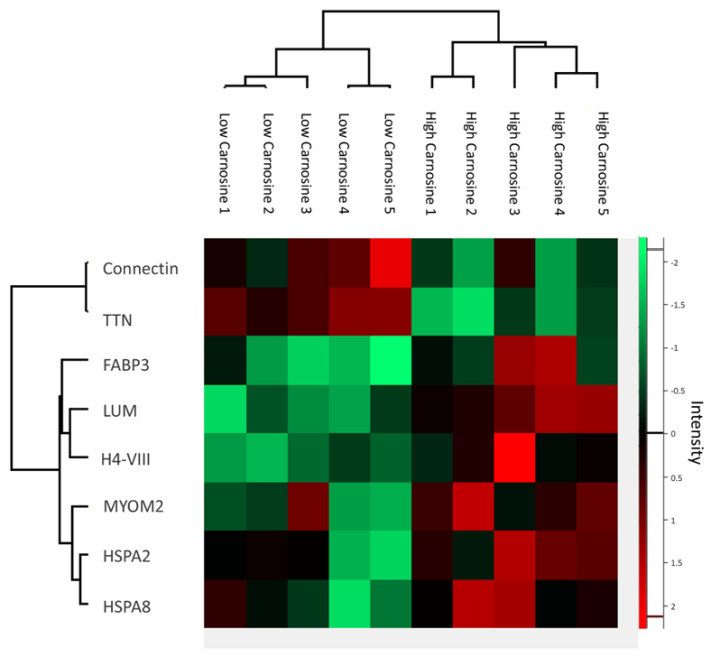
The hierarchical clustering of 8 differential abundant proteins in Korat chickens breast meat between low- and high-carnosine content groups. Each row corresponds to a protein expression level, and red and green colors indicate respectively higher and lower expression relative to average expression across all samples.

A total of 399 proteins were originally identified in the *M. pectoralis major* of the LC and HC groups (Supplementary Table S4). After filtering, the number of expressed proteins was 177, comprising 152 common proteins and 14 or 11 exclusive proteins in the LC and HC groups, respectively (Supplementary Figure S1). The respective exclusive protein names are shown in Supplementary Tables S5 and S6. Among the 152 common proteins, 8 proteins were significantly different in abundance (p<0.05) as shown in Table 1. The hierarchical clustering on protein expression levels of the 8DAPs showed a clear separation between the LC and HC groups (Figure 1). There were 2 low abundance proteins (Titin isoform Ch12 [TTN] and Connectin) and 6 high abundance proteins (heat shock 70 kDa protein 8 [HSPA8], M-protein [MYOM2], fatty acid binding protein 3 [FABP3], lumican [LUM], histone H4 [H4-VIII], and heat shock 70 kDa protein 2 [HSPA2]) in the HC groups. The differential carnosine content in KRC breast meat may related to the physiological and biological processes of these proteins.

### Association of different proteins and their biological and physical parameters

**Figure 2:**
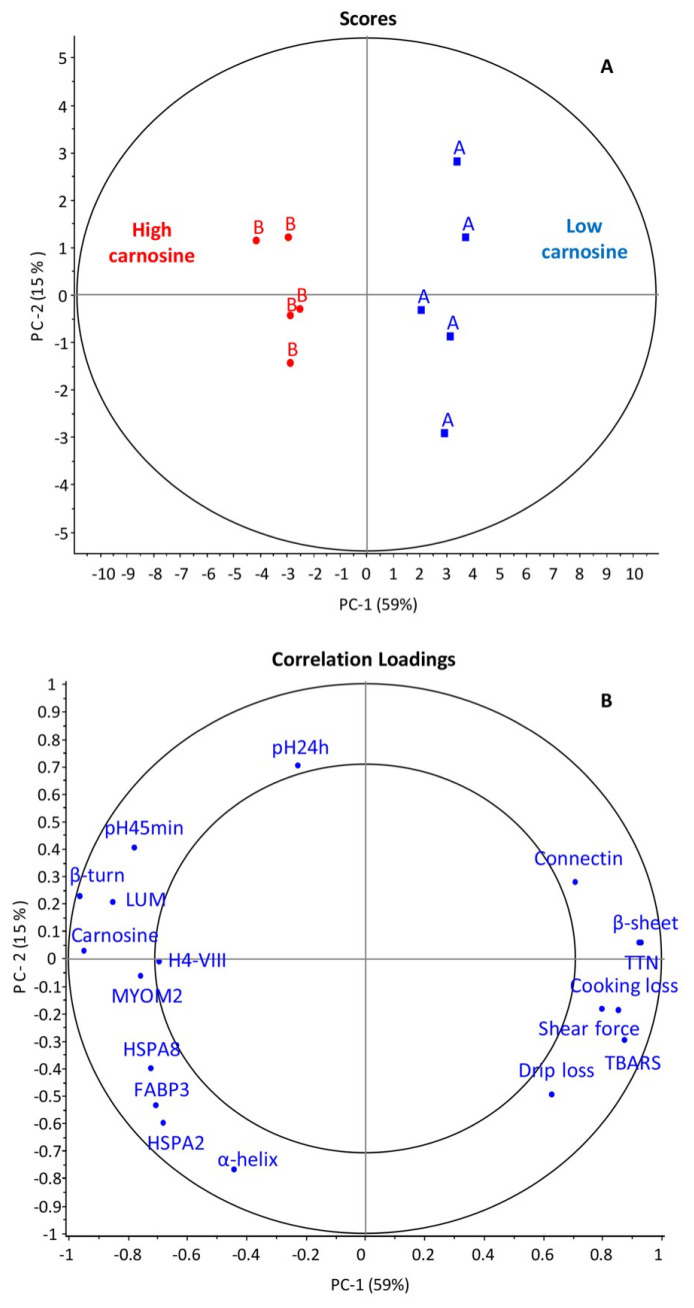
PCA (upper) and correlation loading plot (lower) for 8 DAPs, carnosine content, meat characteristics, and the change of protein secondary structure at 74% total variance for PC1 versus PC2 in Korat chicken breast meat of low-carnosine (A) and high-carnosine (B) as indicated by the outer and inner ellipse, representing 100% and 50% of the variance, respectively. PCA, principal component analysis; DAPs, differential abundant proteins; PC, principal component.

The PCA results showed that the HC group clearly differed from the LC group at a variance greater than 50% identified by the outer and inner ellipses which represents 100% and 50% of the variance, respectively (Figure 2A). Carnosine content; LUM, FABP3, HSPA8, HSPA2, and MYOM2; β-turn; α-helix; and pH_45 min_ positively correlated with each other, whereas they were negatively correlated with TTN, Connectin, β-sheet, drip loss, cooking loss, shear force, and the TBARS values (Figure 2B). The highly abundant protein (LUM, FABP3, HSPA8, HSPA2, and MYOM2) may contribute to differential carnosine content in breast meat by modifying muscle functions and consequently improving meat characteristics.

### Function enrichment analysis

A total of 33 proteins from the combination of the exclusive proteins in the LC (14 proteins) and HC (11 proteins) groups and 8 DAPs were submitted to the functional enrichment analysis for a better understanding of the holistic mechanisms that are responsible for carnosine synthesis (Table 2).

The KEGG pathway analysis revealed that the DAPs (HSPA8 and HSPA2) and the exclusive proteins in the HC group (endoplasmic reticulum resident protein 29 [ERP29] and Protein disulfide isomerase family A, member 6 [PDIA6]) were related in protein processing in the endoplasmic reticulum pathway (FDR<0.05).

We also found the most significantly enriched GO terms that belonged to muscle contraction, protein muscle stability, and protein folding in the biological process category, such as the skeletal muscle thin filament assembly, skeletal muscle myosin thick filament assembly, or cardiac muscle fiber development. The significantly enriched terms for molecular function were composed of structural constituents of muscle and muscle alpha-actinin binding. The proteins involved in cellular components were mainly enriched in muscle fiber composed of striated muscle thin filament, actin cytoskeleton, Z-disc, and M-band.

### Protein-protein interaction network

**Figure 3:**
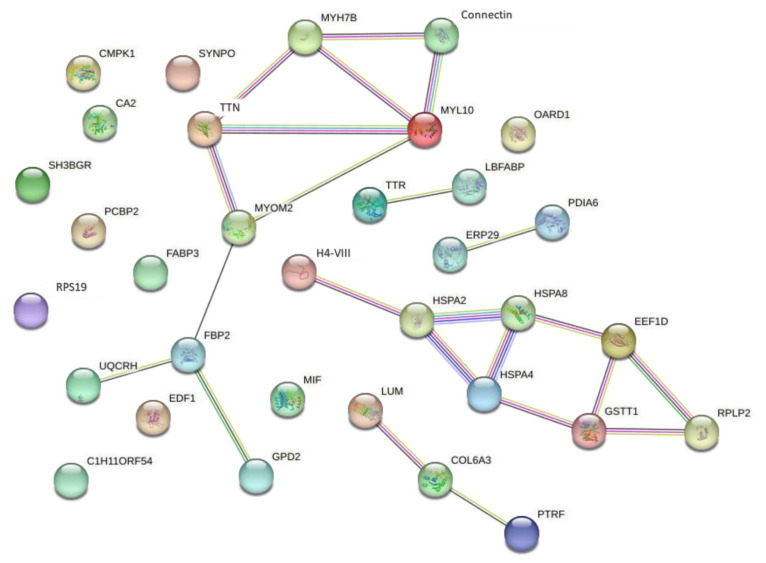
The predicted protein–protein interaction network from the combined protein of DAPs and exclusively of each group. Nodes represent the proteins from the *Gallus gallus* database and lines represent the connections between the proteins (green line, neighborhood; red line, fusion; blue line, co-occurrence; yellow, text mining; black, co-expression; and purple, protein homology). DAPs, differential abundant protein.

The PPI using the STRING platform was performed to analyze the interaction of the 33 proteins, and the results are shown in Figure 3. Two separated networks (PPI enrichment at p-value = 1.55e-0.5) were revealed, and proteins related to stress-responsiveness (H4-VIII, HSPA8, HSPA2, heat shock 70kDa protein 4 [HSPA4], glutathione S-transferase [GSTT1], 60S acidic ribosomal protein P2 [RPLP2], and elongation factor 1-delta isoform [EEF1D]) had pivotal roles in the network. The other set of proteins was related to cytoskeleton proteins (TTN, Connectin, myosin, heavy chain 7B [MYH7B], and myosin, light chain 10, regulatory [MYL10]) and ATP production (fructose-bisphosphatase [FBP2], glycerol-3-phosphate dehydrogenase [GPD2], and cytochrome b-c1 complex subunit 6 [UQCRHL]). Moreover, 3 protein and 2 protein interactions were also found, comprising the connective tissue proteins (LUM, polymerase I and transcript release factor [PTRF], and collagen alpha-3(VI) chain [COL6A3]), ERP29 and PDIA6, and binding proteins (transthyretin [TTR] and fatty acid-binding protein, liver [LBFABP]). Additionally, we found independent proteins, composed of FABP3, UMP-CMP kinase (CMPK1), endothelial differentiation-related factor 1 (EDF1), synaptopodin (SYNPO), macrophage migration inhibitory factor (MIF), O-acetyl-adp-ribose deacetylase 1 (OARD1), poly(rc)-binding protein 2 isoform (PCBP2), ribosomal protein S19 (RPS19), SH3 domain-binding glutamic acid-rich-like protein (SH3BGR), uncharacterized protein (C1H11ORF54), and carbonic anhydrase 2 (CA2). The results showed that carnosine synthesis and its functions were regulated by the reactions of the protein complex network.

## DISCUSSION

In this study, we aimed to describe the global pathways related to carnosine synthesis, molecular functions, and their consequences on muscle structure and functions using a proteomic approach.

The ATPGD1 is a carnosine synthase that ligates β-alanine and histidine to form carnosine, and the sequence corresponds to 100-kDa polypeptide that is present in the most highly purified preparation of chicken carnosine synthase, and the recombinant mouse and human ATPGD1 catalyzes the ATP-dependent synthesis of carnosine and homocarnosine [12]. However, the ATPGD1 related to carnosine synthesis was not found in our study and this might be due to the age of the chickens because the activity of the antioxidant enzymes diminishes as age increases [23]. This fact is supported by the findings of Khumpeerawat et al [10], who found that *ATPGD1* expression linearly decreased when chicken age increases. The age of the chickens used in the current study (70-day-old) might be too old to detect ATPGD1 expression. However, the results of our study describe for the first time the global change of proteins in breast meat with different carnosine contents through dietary supplementation.

Interestingly, ATPGD1 and the pathways related to this protein may not be the only protein or pathway involved in carnosine synthesis, as the proteins found in this study that enriched in the protein processing of the endoplasmic reticulum (ER) of both LC and HC groups (i.e., HSPA8, HSPA2) or the exclusive protein in the HC (i.e., PDIA6, and ERP29) may also be associated with carnosine synthesis. The pathway of protein processed in the ER was revealed in the HC group to play a role in glycosylation, involving the accurate folding of proteins and resisting the action of digestive enzymes [24]. HSPA8 and HSPA2 are members of the heat shock protein 70 family that act as chaperones to control protein folding when cells are exposed to stresses [25]. PDIA6 and ERP29 belong to the family of protein disulfide isomerases that serve as molecular chaperons for protein folding and their maturing into proper tertiary or quaternary structures [26]. In our study, the supplemented β-alanine and L-histidine may have stimulated oxidative stress [27], consequently deteriorating pH homeostasis or proteostasis within the cell [23]. To recover homeostasis and maintain protein function in muscle cells, the pathways, and proteins accordingly function, and ERP29 and PDIA6 proteins may promote the expression of HSPA8 and HSPA2 to combine free β-alanine with L-histidine, causing a carnosine concentration increase in breast muscle.

Additionally, this is the first identification of proteins and their global biological processes, global molecular functions, and cellular components based on carnosine concentration in breast meat of slow-growing chicken through dietary supplementation. All processes are related to maintaining homeostasis in muscles during their functions, including the cardiac muscle. In other words, the results of the cellular components reveal that carnosine functions in the sarcomere, thereby a decreased Titin and Connectin, and an increased MYOM2, resulting in the production of passive force [28]. Furthermore, this preserves the M-band in the middle of the sarcomere [29], within the contractile, leading to the contraction of the muscle. Generally, muscles can function when cells are in homeostasis [30]; however, anaerobic glycolysis during high-intensity muscle contraction leads to a decline in intracellular pH [31] and denaturation, misfolding, and malfunction of proteins [32]. Carnosine acts as a buffer when anaerobic glycolysis occurs in muscles [33] and inhibits free radical activity by providing an electron to a radical molecule, leading to intracellular pH homeostasis [3]. This research finding pointed out that carnosine may be synthesized to restrain environmental extremes as a consequence of glycolytic activity in muscle. Taken together, we can conclude that the maintenance of homeostasis and stress in the skeletal muscle cells are the functions of carnosine in KRC.

Additionally, the results obtained from PCA showed posi tive loading between carnosine content and pH_45 min_ and described the secondary structure of proteins (β-turn and α-helix), proving that maintaining cellular homeostasis is a major function of carnosine. Maintenances of pH, temperature, oxygen concentration, and energy supply homeostasis in living animals are important processes that are deactivated quickly after death, influencing water-holding, tenderness, and meat color [34]. This association explains that carnosine acts as a buffer to maintain intracellular pH homeostasis in early postmortem muscle [3]. Hence, pH_45 min_ was not considerably low, and under the intracellular pH homeostasis, stability of protein structure represented by the high secondary structure of proteins (β-turn and α-helix) was found [35]. A stable protein structure causes an improvement in water-binding ability [36]. These are desirable phenotypes pertaining to meat characteristics.

In this study, the PPI networks revealed that PDIA6 and ERP29 were related proteins. It was supported by a previous study suggesting that PDIA6, ERP29, and heat shock proteins are in the same module since they belong to the family of protein disulfide isomerases [26]. Moreover, the PPI networks revealed an interaction among MYOM2, TTN, MYL10, Connectin, MYH7B, and FBP2, and they help in the maintenance of homeostasis and stress in the skeletal muscle cells. Interestingly, MYL10 and FBP2 or MYH7B are the exclusive proteins found in the LH or HC groups, respectively. Hence, MYL10, FBP2, and MYH7B may have a specific function in the maintenance of homeostasis and stress in the skeletal muscle cells depending on the carnosine levels in the meat.

## CONCLUSION

This study reveals that the pathways of protein processed in the ER and that of the proteins HSPA8, HSPA2, PDIA6, and ERP29 are involved in different carnosine concentrations in the *M. pectoralis major* of the slow-growing KRC. Moreover, the global molecular function of carnosine was also revealed, and carnosine possibly stimulates MYOM2, TTN, MYL10, Connectin, MYH7B, and FBP2 expression to maintain homeostasis and stress in the skeletal muscle cells, which MYL10 and MYH7B function depending on the carnosine levels in the meat. However, carnosine concentration in different ages, genes, proteins related to carnosine synthesis, and meat characteristics needs to be clarified in further studies. Taken together, β-alanine and L-histidine supplementation can increase carnosine content in *M. pectoralis major* without adverse effects on meat quality in slow-growing KRC [14], and the current study suggests that the carnosine synthesis may occur when muscle cells need to recover homeostasis after being interfered with carnosine synthesis precursors, leading to improved muscle function. This evidence provides the confidence to take a step toward genetic enhancement of carnosine synthesis without adversely impacting the molecular function in chickens.

## Figures and Tables

**Table 1:** The differential abundant proteins in the Korat chickens breast meat between low- and high-carnosine content groups

Uniport ID	Gene	Protein names	p-value	FC^1)^
A0A1D5PFJ6	*HSPA8*	Heat shock 70 kDa protein 8	0.042	−1.233
A6BLM7	*TTN*	Titin isoform Ch12 (Fragment)	0.000	1.903
A6BM71	*TTN*	Connectin	0.034	1.272
Q02173	*MYOM2*	M-protein	0.045	−1.222
Q6DRR5	*FABP3*	Fatty acid binding protein 3	0.015	−1.719
P51890	*LUM*	Lumican	0.001	−1.850
P70081	*H4-VIII*	Histone H4	0.021	−1.439
B3VHV2	*HSPA2*	Heat shock 70kDa protein 2	0.035	−1.269

1) FC, fold change ≥1.

**Table 2:** Functional enrichment analysis of differential abundant proteins and proteins exclusively identified in the Korat chickens breast meat between the low- and high-carnosine groups

Term ID	Term description	adj. p-value^1)^	Proteins^2)^
KEGG pathway			
gga04141	Protein processing in endoplasmic reticulum	0.034	HSPA8, HSPA2, PDIA6, ERP29
Biological process			
GO:0030240	Skeletal muscle thin filament assembly	0.007	TTN, Connectin
GO:0030241	Skeletal muscle myosin thick filament assembly	0.007	TTN, Connectin, MYOM2
GO:0048739	Cardiac muscle fiber development	0.007	TTN, Connectin
GO:0055003	Cardiac myofibril assembly	0.007	TTN, Connectin, MYOM2
GO:0045214	Sarcomere organization	0.027	TTN, Connectin, MYOM2
GO:0055008	Cardiac muscle tissue morphogenesis	0.038	TTN, Connectin, MYOM2
GO:0048769	Sarcomerogenesis	0.039	TTN, Connectin, MYOM2
GO:0035995	Detection of muscle stretch	0.047	TTN, Connectin, MYOM2
GO:0043933	Protein-containing complex subunit organization	0.047	TTN, Connectin, MYOM2, 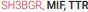 , H4-VIII, 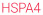 , HSPA8
Molecular function			
GO:0008307	Structural constituent of muscle	0.035	TTN, Connectin, MYOM2
GO:0051371	Muscle alpha-actinin binding	0.027	TTN, Connectin, MYOM2
Cellular component			
GO:0005865	Striated muscle thin filament	0.021	TTN, Connectin, MYOM2
GO:0015629	Actin cytoskeleton	0.021	TTN, Connectin, MYOM2
GO:0030018	Z disc	0.021	TTN, Connectin, MYOM2, 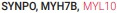
GO:0031430	M band	0.021	TTN, Connectin, MYOM2, 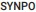

KEGG, Kyoto encyclopedia of genes and genomes; FDR, false discovery rate.

1) FDR-adjusted p-value <0.05 [20].

2) Bold characters represent the exclusive protein in the high-carnosine group and red characters present the exclusive protein in the low-carnosine group.

## SUPPLEMENTARY MATERIAL

**Supplementary Table S1**. Ingredient and nutrient composition of experimental diets in different growing phases

**Supplementary Table S2**. Meat characteristics and carnosine content in meat published by Suwanvichanee et al [14]

**Supplementary Table S3**. Data used for principal component analysis

**Supplementary Table S4**. Proteins identified by LC-MS/MS analysis in breast meat samples from Low- and high-carnosine content of Korat chicken

**Supplementary Table S5**. The list of proteins exclusively identified in the low-carnosine group

**Supplementary Table S6**. The list of proteins exclusively identified in the high-carnosine group

**Supplementary Figure S1**. Venn Diagram representing the number of common proteins (center) and exclusive proteins identified in the KRC breast meat in low- (left) and high-carnosine content (right)
