# Supplementary material for: Revealing the global mechanism related to carnosine synthesis in the pectoralis major of slow-growing Korat chickens using a proteomic approach
Source: Anim Biosci. 2024 Aug 14;37(10):1692–701. doi: 10.5713/ab.24.0119 (PMC11366509; doi:10.5713/ab.24.0119)
Supplement: Supplementary file 1 [file ab-24-0119-Supplementary-Table-1.pdf]

Table S1. Ingredient and nutrient composition of experimental diets in different growing phases

	Grower (22 to 42 days)[1]		Finisher (43 to 70 days)[1]	
	A	B	A	B
Ingredients (kg)				
Corn	59.40	56.64	67.30	64.44
Soybean meal (44% CP)	30.80	28.78	26.30	26.70
Full fat soybean	2.42	5.20	0.00	0.00
Rice bran oil	4.00	4.50	3.04	4.00
L-Lysine	0.18	0.18	0.19	0.19
DL-Methionine	0.21	0.21	0.14	0.14
Salt	0.35	0.35	0.35	0.35
Calcium carbonate	1.42	1.42	1.20	1.20
MDCP (P21)	1.02	1.02	1.28	1.28
Premix[2]	0.20	0.20	0.20	0.20
β-Alanine	0	1.00	0	1.00
L-Histidine	0	0.50	0	0.50
Total (kg)	100	100	100	100
Calculated composition (%)				
ME (kcal/kg)	3,113	3,111	3,112	3,110
Crude protein, %	19.39	19.29	17.08	17.04
Crude fiber, %	3.60	3.55	3.32	3.29
Ether extract, %	6.94	7.82	5.81	6.66
Calcium, %	0.90	0.90	0.86	0.86
Total phosphorus, %	0.57	0.56	0.60	0.59
Histidine	0.45	0.98	0.37	0.87

[1]Treatment groups are A (Low-carnosine group) and B (High-carnosine group)

[2]Premix (0.5%) provided the following per kilogram of diet: 15,000 IU of vitamin A, 3,000 IU of vitamin D3, 25 IU of vitamin E, 5 mg of vitamin K3, 2 mg of vitamin B1, 7 mg of vitamin B2, 4 mg of vitamin B6, 25 ug of vitamin B12, 11.04 mg of pantothenic acid, 35 mg of nicotinic acid; 1 mg of folic acid, 15 µg of biotin, 250 mg of choline chloride, 1.6 mg of Cu, 60 mg of Mn, 45 mg of Zn, 80 mg of Fe, 0.4 mg of I and 0.15 mg of Se.
