# Supplementary material for: Revealing the global mechanism related to carnosine synthesis in the pectoralis major of slow-growing Korat chickens using a proteomic approach
Source: Anim Biosci. 2024 Aug 14;37(10):1692–701. doi: 10.5713/ab.24.0119 (PMC11366509; doi:10.5713/ab.24.0119)
Supplement: Supplementary file 2 [file ab-24-0119-Supplementary-Table-2.pdf]

Table S2. Meat characteristics and carnosine content in meat published by Suwanvichanee et al. [14]

Parameter	A	B	SEM[2]	P-value
Carnosine (μg/g)	2,756.6	4,212.5	68.44	<0.001
pH_{45min}	5.27	5.40	0.03	0.008
pH_{24h}	5.36	5.38	0.01	0.400
Drip loss (%)	13.64	12.61	0.38	0.096
Cooking loss (%)	25.83	24.05	0.33	0.005
Shear force (kg)	3.31	2.81	0.09	0.005
TBARS (mg/kg)	0.22	0.10	0.01	<0.001
β-sheet (1630 cm^{-1})	22.24	18.72	0.40	<0.001
α-helix (1644 cm^{-1}, 1655 cm^{-1})	49.44	50.74	0.537	0.189
β-turn (1670 cm^{-1})	15.61	17.63	0.199	<0.001

A = Low-carnosine group and B = High-carnosine group
