# Supplementary material for: Revealing the global mechanism related to carnosine synthesis in the pectoralis major of slow-growing Korat chickens using a proteomic approach
Source: Anim Biosci. 2024 Aug 14;37(10):1692–701. doi: 10.5713/ab.24.0119 (PMC11366509; doi:10.5713/ab.24.0119)
Supplement: Supplementary file 3 [file ab-24-0119-Supplementary-Table-3.pdf]

Table S3. Data used for principal component analysis

treatment	β-sheet	α-helix	β-turn	pH45min	pH24h	Driploss	Cookingloss	Shearforce	TBARS	Carnosine	HSPA8	TTN	Connectin (TTN)	MYOM2	FABP3	LUM	HIST2H4B	HSPA2
LC1	22.27982	51.68611	15.14891	5.23	5.31	14.72	25.8	3221.3	0.25	2900.78	0.382483	0.716886	0.182589	-0.672906	-0.214109	-1.81096	-1.29018	-0.0314227
LC2	23.29011	49.14877	15.26571	5.25	5.34	13.55	25.57	3667.3	0.26	2639.32	-0.124321	0.300063	-0.331259	-0.503844	-1.29995	-0.68632	-1.52791	0.0958876
LC3	20.2908	50.30253	16.18901	5.23	5.36	12.82	25.57	3422.1	0.22	2590.41	-0.484918	0.596945	0.604638	0.915373	-1.75305	-1.17696	-0.883973	0.0264053
LC4	22.35245	49.1199	15.49487	5.33	5.37	13.55	26.97	3174.68	0.2	2744.84	-1.85188	1.0986	0.788236	-1.33417	-1.53589	-1.36453	-0.490953	-1.48829
LC5	23.00888	47.96051	15.94991	5.3	5.41	13.55	25.25	3086.75	0.19	2907.73	-1.00306	1.11665	1.93678	-1.45893	-2.2784	-0.490837	-0.814727	-1.7739
HC1	17.99406	51.76334	17.41496	5.41	5.38	13.45	23.94	2993.7	0.11	4472.61	0.0330393	-1.53287	-0.473494	0.456854	-0.120483	0.0955086	-0.314131	0.314296
HC2	18.85748	49.41922	18.35892	5.51	5.35	11.52	23.02	2587.4	0.12	4072.91	1.50395	-1.85703	-1.33926	1.59723	-0.515175	0.247973	0.257681	-0.215419
HC3	18.68936	51.14158	17.51827	5.34	5.35	12.99	24.84	2635.5	0.14	4091.06	1.36218	-0.469651	0.38741	-0.150409	1.27458	0.787327	2.26587	1.49215
HC4	19.16715	51.23497	17.1897	5.35	5.41	13.55	24.81	2901.2	0.07	4199.57	-0.0359314	-1.33002	-1.33051	0.349252	1.43177	1.3247	-0.0944956	0.849378
HC5	18.87326	50.11573	17.64279	5.37	5.39	11.56	23.63	2932.1	0.07	4226.21	0.218466	-0.49661	-0.425131	0.801552	-0.55663	1.26314	0.0747251	0.730923
