# Supplementary material for: Revealing the global mechanism related to carnosine synthesis in the pectoralis major of slow-growing Korat chickens using a proteomic approach
Source: Anim Biosci. 2024 Aug 14;37(10):1692–701. doi: 10.5713/ab.24.0119 (PMC11366509; doi:10.5713/ab.24.0119)
Supplement: Supplementary file 4 [file ab-24-0119-Supplementary-Table-4.pdf]

Table S4. Proteins identified by LC-MS / MS analysis in breast meat samples from Low- and high-carnosine content of Korat chicken.

Uniprot	Protein name	Gene name	Peptides	Razor +	Unique	Sequenc	Unique	Unique s	Mol. weig	Q-value	Score	Intensity	MS/MS cc	LFQ intensi	LFQ intens	LFQ inter	LFQ inten	LFQ inte	LFQ intensi	LFQ inten	LFQ intens	LFQ intens	LFQ intensity High5
A0A0A0MQ61;C	Glutathione S-transfera	GSTAL1;GSTAL2;C	2	2	2	12.7	12.7	12.7	25.222	0	4.3733	2935.2	3	0	0	0	0	0	0	0	665.7	0	0
P80226;A0A140	Fatty acid-binding prot	FABP1;LBFABP	2	2	2	16.7	16.7	16.7	14.21	0	3.2026	69152	4	0	0	0	0	0	22148	0	77678	0	0
A0A1D5PXG0;E	Heterogeneous nuclear	HNRNPK	4	4	1	13.4	13.4	4	47.102	0	10.98	25094	13	0	0	5363.2	0	0	0	0	0	0	0
A0A1L1RY35;A	ATP synthase alpha sul	ATP5A1;ATP5A1W	24	24	2	47.8	47.8	7.2	58.41	0	320.82	3015600	109	392960	307970	447650	217770	126710	352910	415190	376370	226940	294610
Q5ZMU9;A0A1I	Uncharacterized protei	TERAL2;VCP vcp F	14	14	14	21.6	21.6	21.6	89.324	0	66.387	203870	38	11570	26559	25967	0	0	15638	48873	25154	22170	14286
P17785;A0A1C9	Annexin;Annexin A2 (A	ANXA2;ANXA2 AI	1	1	1	4.7	4.7	4.7	38.64	0	4.1003	1368.9	3	0	1095.3	0	0	0	0	0	0	0	0
A0A1D5NT92	Uncharacterized protei	MYOT	3	3	3	7.8	7.8	7.8	57.366	0	7.1834	175790	9	0	0	0	0	16089	18589	23634	31732	33091	0
A0A1D5NTD0;A	Uncharacterized protei	ALPK3	2	2	2	1.5	1.5	1.5	166.76	0	3.4826	1976.3	3	0	0	0	0	0	0	0	0	0	838.26
A0A3Q2TVA9;A	Complement factor D		1	1	1	3.1	3.1	3.1	49.504	0.00783	2.6504	30872	0	0	0	0	0	0	0	0	13808	0	0
A0A1D5NTS2	Uncharacterized protei	CA3A	17	17	17	60.7	60.7	60.7	29.722	0	199.13	1663600	66	26995	55623	450040	11098	811960	140770	84011	40984	219490	0
E1BYN7;A0A1D	Uncharacterized protei	VDAC1	3	3	3	13.4	13.4	13.4	30.706	0	11.737	81226	9	0	16063	0	9965.8	0	0	0	0	10917	0
A0A1D5NUM3;A	Uncharacterized protei	AAMDC	1	1	1	11.5	11.5	11.5	13.123	0	10.008	30955	5	0	0	0	0	8218.1	0	0	0	0	0
A0A1D5NUR5	Uncharacterized protei	SNTA1	2	2	2	4.8	4.8	4.8	51.678	0.00787	2.6999	13166	4	0	0	0	0	0	0	5344.8	0	0	0
A0A1L1RZD1;Q	Uncharacterized protei	SRSF3;SRSF7;SRSI	2	2	2	23	23	23	15.621	0	4.0312	986.24	2	0	0	0	610.56	0	0	0	0	0	0
A0A1D5NVM2	NADH dehydrogenase	NDUFA2	2	2	2	25	25	25	11.071	0	4.4105	44447	6	0	0	0	0	0	0	0	0	0	15117
P02552;A0A1D5	Tubulin alpha chain;Tu	LOC425049;TUBA1	4	1	1	12.6	3.6	3.6	45.9	0.00279	3.1158	1936.2	1	0	0	0	0	0	0	0	0	0	346.33
F1NNP6;A0A1D	Uncharacterized protei	GLRX3	2	2	2	11.3	11.3	11.3	36.574	0	4.6998	789.9	2	0	0	0	1143.7	0	0	0	0	0	0
A0A1D5NW68	Serum albumin	ALB	34	34	2	57.3	57.3	1.6	64.004	0	237.16	7065500	241	546650	848840	691620	872900	610090	670550	858220	1023600	775400	671710
A0A1D5NWH9;(	Rous sarcoma virus trai	YBX3	1	1	1	5.2	5.2	5.2	30.873	0	7.2855	33816	4	0	0	0	0	0	0	0	0	9437.8	0
Q90602;A0A1D5	Heterogeneous nuclear	ribonucleoprotein A/	4	4	4	13.9	13.9	13.9	31.861	0	10.521	24163	11	0	0	0	0	0	0	4156.2	0	0	0
A0A1D5NXK7;A	Uncharacterized protei	PFDN5	2	2	2	18.1	18.1	18.1	17.259	0	4.881	6585.7	4	0	0	0	4916.3	0	1157.1	0	0	2103.6	0
Q5ZI93;A0A1L1	Uncharacterized protei	TPD52L2;TPD52L2	2	2	2	9.8	9.8	9.8	22.181	0	4.9331	9054.3	4	0	0	0	0	0	0	0	0	2576.8	0
A0A1L1RJP8;A0	Tubulin alpha chain;Tu	TUBA1C	4	4	1	10.4	10.4	3	55.274	0	16.108	144480	12	15185	21520	18197	12212	8931.3	9332.2	24935	0	0	14288
A0A1D5NXY4	Uncharacterized protei	BANF1	6	6	6	53.3	53.3	53.3	10.057	0	31.214	92171	36	11616	10166	7979.3	13742	18189	8962.5	7636.5	20696	12586	5852.6
A0A1D5NYC2	Fast-white myosin heav	AMHC1;MYH1E	190	3	0	68.1	1.7	0	223.13	0	22.726	351970	25	16200	41616	40894	79286	100900	27138	30817	42352	63008	32216
A0A1D5NYF3;C	Adipocyte fatty acid bi	AFABP	8	8	7	64.4	64.4	64.4	14.921	0	52.205	72033	8	0	0	0	0	0	0	0	85552	0	0
A0A1D5NYX1;F	Eukaryotic translation i	EIF1;EIF1 SUI1	3	3	2	14.2	14.2	6.6	22.39	0	7.812	30986	7	0	0	0	14579	0	0	0	14476	0	0
Q98916;A0A1D5	Slow muscle troponin T	sTnT TNNT1;TNNT	6	6	6	21.1	21.1	21.1	31.867	0	21.789	86877	7	0	0	0	0	52444	0	0	0	75203	0
A0A1D5NZ30;P	Nucleolin;Nucleolin (P	NCL	2	2	2	3.2	3.2	3.2	75.399	0	6.377	5524.1	1	0	0	0	0	0	0	0	5373.7	0	0
F1NYE5;Q5F3L	Uncharacterized protei	RCJMB04_14f6;SE	3	3	3	10.4	10.4	10.4	45.477	0	5.1821	70721	3	0	0	0	0	0	0	0	0	21570	0
A0A1D5NZY9;C	Myosin heavy chain;Sl	MYH7B;SM2;SSMI	25	7	5	10.3	4	2.9	224.26	0	35.536	5269500	22	0	0	0	0	0	0	745420	0	828660	560940
A0A1D5P061;A0	Phosphorylase b kinase	PHKA1	2	2	2	2.1	2.1	2.1	134.07	0	4.0459	11134	5	0	0	0	0	0	0	1073.6	0	0	0
A0A1L1RP39;Q	UTP--glucose-1-phosp	RCJMB04_8o6;UGI	7	7	7	18.3	18.3	18.3	55.633	0	29.591	169820	13	0	15509	0	0	0	0	0	0	0	0
A0A1D5P0B5	Uncharacterized protei	KLHL41	3	3	3	5	5	5	68.552	0	4.9105	45897	2	3366.7	0	0	0	0	0	0	0	0	0
A0A1D5PH14;A	Dynein light chain road	DYNLRB1;DYNLR	1	1	1	9.5	9.5	9.5	10.78	0	3.4933	0	1	0	0	0	0	0	0	0	0	0	0
A0A1D5P1R0	Junctophilin 1		4	4	4	3.7	3.7	3.7	87.919	0	17.418	103900	10	0	0	0	0	0	0	0	0	0	16651
A0A1D5P1U2	Pyruvate dehydrogenas	PDHB	4	4	4	14.2	14.2	14.2	38.877	0	11.138	4790.2	4	0	0	1766.4	0	0	0	0	0	0	0
P04268;A0A1D5	Alpha tropomyosin of b	alpha-fTM;TPM1	51	51	14	78.9	78.9	19.7	32.765	0	323.31	97373000	614	12661000	9833900	1.1E+07	7252000	9E+06	11411000	11528000	11385000	8845600	1.2E+07
Q90ZK7;A0A1D	Peptidylprolyl isomeras	FKBP25	5	5	5	26.9	26.9	26.9	25.031	0	20.506	194540	24	0	28709	18268	47454	36327	23263	0	41499	38806	0
A0A1D5PI24;A0	Uncharacterized protei	NFATC1	1	1	1	2.2	2.2	2.2	80.134	0.0101	2.5209	0	1	0	0	0	0	0	0	0	0	0	0
A0A1D5P470	Glutathione S-transferase (EC 2.5.1.18)		2	2	2	10	10	10	25.449	0	4.5388	40871	6	13382	0	13060	0	0	0	0	0	0	0
A0A1D5P525;Q	Fast myosin heavy chai	MYH1C	136	23	2	51.5	11.8	0.6	223.24	0	212.64	1184300	84	76754	67069	163040	123850	364700	92936	79299	143400	214770	104490
A0A1D5P5M6;E	Uncharacterized protei	ES1ML2	10	10	9	47.8	47.8	44.5	26.794	0	36.413	650860	26	67556	70444	47564	60438	123320	50428	64433	119760	68845	36140
A0A1D5P5R0;P	Heat shock cognate pro	HSP90AA1 HSPCA	9	9	9	12.7	12.7	12.7	83.875	0	32.347	176860	25	0	30917	39270	0	0	0	46690	0	0	0
A0A1D5P5Z0	Tubulin alpha chain	TUBA8A	3	1	1	8.1	3.4	3.4	50.107	0	4.6402	3631.7	1	0	0	0	0	0	0	1925	0	0	0
Q910C5;A0A1D	Atrial myosin heacy ch	MYH7	29	1	0	10.2	0.6	0	221.8	0	109.21	664770	21	0	0	83822	88886	106400	0	0	90201	132990	0
F1P4X4;A0A1D	Uncharacterized protein		1	1	1	3.3	3.3	3.3	51.899	0.00785	2.6811	0	1	0	0	0	0	0	0	0	0	0	0
A0A1D5P6V5	Perilipin	PLIN4	2	2	2	5.4	5.4	5.4	45.388	0	4.5074	8556.6	4	0	0	0	0	10995	0	0	0	0	0
Q90706;A0A1D5	CLE7;RNA transcriptic	CLE7	5	5	5	30.5	30.5	30.5	27.389	0	13.441	9026.5	11	0	0	0	0	5017.8	0	0	0	1875.8	0
A0A3Q2UJ64;D	Cytochrome b-c1 comp	UQCRB	3	3	3	30.8	30.8	30.8	13.143	0	8.3869	159020	8	34143	0	0	54378	0	0	0	0	51749	0
A0A1D5P810	Uncharacterized protei	ATP5O	8	8	8	39.8	39.8	39.8	26.639	0	23.579	378410	24	0	42886	54525	53107	55184	41894	45619	46931	47154	37868
A0A1D5P893;A	Uncharacterized protei	LOC426023	2	2	2	6.7	6.7	6.7	34.832	0	6.6903	66807	7	22294	17475	0	0	0	0	0	0	0	0
F1NUF6;B8XA3	Disintegrin and metallo	ADAM22	1	1	1	2.5	2.5	2.5	85.944	0.01008	2.507	948300	4	0	0	0	0	0	0	154980	0	0	0
A0A1D5P8F9	Uncharacterized protei	CNPY2	1	1	1	8.4	8.4	8.4	21.189	0.00777	2.6074	0	2	0	0	0	0	0	0	0	0	0	0
A0A1D5P8I3	Uncharacterized protei	SFPQ	1	1	1	2.3	2.3	2.3	69.532	0.00543	2.9219	8841.4	4	0	0	0	0	0	0	0	0	0	2394.2
A0A1D5P8I7;Q	Isocitrate dehydrogenas	IDH2;RCJMB04_7e	6	6	6	17.5	17.5	17.5	50.482	0	20.082	95225	24	5509.2	11528	30005	9622.7	0	6326.4	3551.3	0	0	12825
A0A1D5P8K2;A	Small heat shock protei	HSPB2	3	3	3	23.1	23.1	23.1	19.721	0	9.4195	27604	8	0	0	0	0	0	0	0	0	0	8415.9
A0A1D5P8X3;A	Uncharacterized protei	PDLIM5;RCJMB04	18	18	18	79.4	79.4	79.4	23.363	0	309.31	7783000	168	829280	713510	682420	962410	968510	903150	843980	980410	853200	817900
Q5ZI23-2;A0A3	Hydroxyacylglutathion	HAGH;HAGH RCJI	1	1	1	4.6	4.6	4.6	29.031	0	6.7331	5230.2	4	0	0	0	0	0	0	0	0	1138.1	0

A0A1D5P909	Uncharacterized protei	LDB3	21	21	21	63.3	63.3	63.3	31.17	0	323.31	17146000	251	1258100	1610500	1687100	2270400	2E+06	1769600	1846700	2379800	2218300	1774500
Q5ZKG5;A0A1D	Low molecular weight	ACP1;ACP1 RCJMI	5	5	5	35.4	35.4	35.4	18.196	0	11.828	71803	9	15203	0	0	0	15735	0	0	28904	20998	14104
A0A1D5PAA8;Q	Glycogenin 1;Uncharac	RCJMB04_16h16	7	7	7	28.6	28.6	28.6	37.2	0	82.904	303880	29	0	40882	48021	33208	34209	34127	0	23854	13623	64010
A0A1D5PAN0	Proline and arginine rich end leucine rich re		5	5	5	15.6	15.6	15.6	48.976	0	12.957	43023	18	0	0	0	8774.8	0	6015.5	5543.9	0	11838	11143
A0A1D5PAU7	Uncharacterized protei	ACY1	2	2	2	6.8	6.8	6.8	45.909	0	6.7064	17077	3	0	0	0	0	0	0	4523.4	0	0	0
A0A1D5PBD2	Uncharacterized protein		2	2	2	10.9	10.9	10.9	10.456	0	3.7861	122230	2	0	0	0	0	74984	0	0	0	0	0
A0A1D5PCL3;A	Uncharacterized protei	MYBPC1	7	5	5	7.2	5.8	5.8	128.49	0	12.451	54381	7	0	0	37121	0	0	0	0	0	0	0
A0A1D5PBY3;A	Uncharacterized protei	PRDX3	6	6	6	23.5	23.5	23.5	25.761	0	24.988	344500	36	47500	40092	28166	43982	40512	30564	25482	83497	37733	0
A0A1D5PC99;Q	Electron transfer flavop	RCJMB04_23a9	2	2	2	7.7	7.7	7.7	34.217	0	3.1954	4959.2	5	0	0	0	0	0	1525.1	0	0	0	0
A0A1D5PDE6;P	Myristoylated alanine-r	MARCKS	5	5	5	24.2	24.2	24.2	27.643	0	39.527	127200	18	62320	0	0	0	0	24513	0	16643	12467	0
A0A1D5PDV6	Uncharacterized protei	RPS19	3	3	3	20.7	20.7	20.7	16.074	0	7.6685	64850	9	0	0	18261	22814	0	0	0	0	0	0
A0A1L1RLN6;P	Myosin light chain 6;M	MYL6	2	2	2	14	14	14	16.838	0	3.9653	6678.5	2	5864.5	0	0	0	0	0	0	0	0	0
F1NX83;A0A1D	Uncharacterized protei	AGL	12	12	12	10.7	10.7	10.7	174.43	0	41.761	166890	22	8217.1	0	21061	0	13226	0	26789	0	0	20683
P02611;A4UNW	Myosin light chain 2;M	MYL10;MYL2;Myl	5	5	5	32.7	32.7	32.7	18.789	0	41.63	40136	7	0	0	16825	0	20479	0	0	0	0	0
A0A1D5PYK0;A	Heat shock cognate 71	HSPA8;HSPA8 HS	29	29	18	49.4	49.4	34	69.114	0	298.81	4907900	241	539110	481590	444440	327830	396020	498760	692000	670500	491160	519780
A0A1D5PGA2	Cyclic nucleotide gated channel beta 3		1	1	1	2.3	2.3	2.3	76.182	0	4.5389	6514700	12	0	546120	0	0	506350	0	221300	0	3775400	2516600
A0A3Q2UD12;A	Collagen alpha-3(VI) c	COL6A3	9	9	9	4.2	4.2	4.2	326.28	0	35.326	54769	16	4184.6	0	5850.4	0	0	0	0	0	0	0
A0A1D5PH32;A	Gelsolin;Gelsolin (Acti	GSN	3	3	3	6	6	6	80.979	0	6.7829	74047	6	0	0	0	0	0	0	0	39351	0	0
Q5ZHW5;A0A1	Proteasome subunit alp	PSMA2;RCJMB04_	1	1	1	24.7	24.7	24.7	9.1344	0.00539	2.897	1942.2	2	0	0	0	1551.3	0	0	0	0	0	0
A0A1D5PI06	Uncharacterized protei	NDUFV2	1	1	1	4.1	4.1	4.1	26.893	0.00765	2.5478	2082.3	3	0	0	0	0	0	0	0	0	0	999.31
A0A1D5PI87;A0	Hydroxysteroid 17-beta dehydrogenase 10		2	2	2	5.3	5.3	5.3	43.384	0	4.2976	2146.2	3	0	0	735.69	0	0	0	0	0	0	0
A0A1D5PTS6;A	Protein-L-isoaspartate (	PCMT1L	2	2	2	6.1	6.1	6.1	26.768	0	3.6047	6406.4	2	0	0	0	5675.7	0	0	0	0	0	0
Q9I9K4;A0A1D	Nidogen 1;Nidogen 1 (Fragment)		6	6	6	14.3	14.3	14.3	52.11	0	14.817	51652	11	0	0	0	0	0	0	15801	0	0	0
Q5ZJ70;A0A1D	Uncharacterized protei	PDCD6IP;RCJMB0	2	2	2	2.5	2.5	2.5	97.314	0	4.0969	31411	4	0	0	0	0	0	0	0	0	0	7419.1
A0A1D5PMT8	Uncharacterized protei	RPLP2	4	4	4	45.2	45.2	45.2	11.842	0	15.613	56779	10	18022	0	0	16287	0	0	0	0	0	0
A0A1D5PN46	Fructose-1,6-bisphosph	FBP2	5	5	5	19.1	19.1	19.1	37.102	0	38.055	225980	20	39389	26557	49654	0	21913	0	0	0	0	0
A0A1D5PN69	Uncharacterized protein		1	1	1	4.6	4.6	4.6	54.95	0.00761	2.5429	27080	1	0	0	0	0	0	0	0	0	38321	0
A0A1I7Q419;A0	40S ribosomal protein	RPS12	2	2	2	16.5	16.5	16.5	13.529	0	4.1179	9848.8	3	0	0	0	0	0	0	0	0	0	4773.1
Q5F405;A0A1D	Uncharacterized protei	RCJMB04_3o20;UI	2	2	2	13.8	13.8	13.8	17.066	0	4.4005	186150	9	0	0	0	22427	0	0	36487	0	0	34737
A0A1D5PPF8	Uncharacterized protei	HSPA4	5	5	5	5.6	5.6	5.6	94.239	0	12.172	26202	10	0	9314.2	0	9842.2	0	0	0	0	0	0
A0A1D5PPL7;A	Uncharacterized protei	EEF1D	3	3	3	15.5	15.5	15.5	32.325	0	13.163	42803	7	0	10158	8477.2	15175	0	0	0	0	0	0
A0A1D5PQ96;Q	3-hydroxyisobutyrate d	HIBADH;HIBADH	2	2	2	5	5	5	31.781	0	5.9127	0	2	0	0	0	0	0	0	0	0	0	0
A0A3Q2U5Z2;A	Spectrin alpha chain, n	SPTAN1;SPTAN1 S	3	3	3	1.9	1.9	1.9	281.56	0	8.9183	2161.3	3	0	0	0	0	2509	0	0	0	0	0
Q5ZJ47;A0A1D	Aspartyl aminopeptidas	RCJMB04_20m7	1	1	1	2.5	2.5	2.5	51.86	0	3.2357	14579	2	0	0	0	0	0	0	6224	0	0	0
A0A1D5PRI6	Neural precursor cell expressed, developme		1	1	1	16.5	16.5	16.5	9.591	0.00276	3.0085	2100.7	2	0	0	0	0	0	0	0	0	0	879.27
A0A1D5PRT3	Uncharacterized protei	SYNPO2L	7	7	7	9.2	9.2	9.2	115.55	0	15.621	184450	15	0	26602	25966	28546	31342	23222	29721	0	41469	0
Q90705;A0A1D	Elongation factor 2 (EF	EEF2	11	11	11	14.2	14.2	14.2	95.377	0	29.677	287910	36	18515	31417	45402	0	0	48595	55356	0	31399	31596
A0A1D5PS70	PDGFA associated protein 1		1	1	1	9.2	9.2	9.2	21.129	0	3.4086	13584	6	0	0	0	0	0	0	0	0	0	1410.3
A0A1D5PSD3	Uncharacterized protein		1	1	1	1.2	1.2	1.2	83.797	0.00535	2.8364	2788500	13	0	0	0	0	0	0	0	0	0	213770
F1N9Z6;A0A1D	Myomesin;Uncharacter	MYOM1	34	34	34	21.9	21.9	21.9	177.33	0	323.31	1940800	132	180130	173900	255780	208640	128530	197200	253210	226880	217440	183290
A0A1D5PTF1;A	Uncharacterized protei	SPEG	1	1	1	0.4	0.4	0.4	362.41	0	4.5466	1497.3	4	0	0	0	0	2908.8	0	0	0	0	0
A0A1D5PUI6	Uncharacterized protei	LOC107049545	3	3	3	25.4	25.4	25.4	13.984	0	4.6321	131020	8	0	0	23949	0	0	0	0	0	0	0
Q5F3G9;A0A1D	Dihydrolipoamide acet	PDHX;RCJMB04_1	1	1	1	3.8	3.8	3.8	53.219	0.00529	2.8056	19338	1	0	0	0	0	0	0	0	0	0	10662
A0A1D5PUM7;I	Uncharacterized protein		40	40	40	29.4	29.4	29.4	233.15	0	230.11	1975600	136	133620	248340	198900	165860	196410	219620	242950	237410	262040	195230
A0A1D5PUR6	Uncharacterized protei	MYH1A	134	2	0	51.7	1.4	0	222.91	0	4.0366	29430	4	0	0	0	0	11084	0	0	0	0	0
A0A3Q2U0R8;A	Uncharacterized protei	HNRNPA2B1;HNR	6	6	6	20.2	20.2	20.2	34.625	0	19.991	79816	22	10604	9999.7	0	18197	0	14188	9877	24766	0	0
A0A1L1RUX2;A	Eukaryotic initiation fa	EIF4A2;EIF4A2 RC	2	2	2	7	7	7	42.389	0	4.1642	36154	2	0	0	0	0	0	0	0	0	4737	0
P20785;A0A1D5	Collagen alpha-1(VI) c	COL6A1	3	3	3	3.5	3.5	3.5	107.98	0	9.1459	96293	6	0	28845	0	0	0	0	0	0	0	0
Q6B344;E1BWX	Annexin	ANXA6	8	1	1	13.5	2.4	2.4	75.226	0.00279	3.0445	12148	1	0	0	10000	0	0	0	0	0	0	0
P02457;A0A1D5	Collagen alpha-1(I) cha	COL1A1	5	5	4	4.5	4.5	4.5	137.5	0	222.2	534520	37	48108	0	24854	201100	40866	54257	57999	148720	140680	0
A0A1I7Q403	Ubiquitin-conjugating	UBE2V1	3	1	1	19.7	8.2	8.2	16.463	0.01	2.4685	0	1	0	0	0	0	0	0	0	0	0	0
P27731;A0A1I7	Transthyretin;Transthy	TTR	3	3	3	22	22	22	16.309	0	11.442	153470	11	0	0	0	0	0	0	0	42062	46234	24835
Q5ZLV5;F1P463	Carnosine dipeptidase	RCJMB04_4l9	2	2	2	5.3	5.3	5.3	53.078	0	3.2603	2497.1	2	0	1998	0	0	0	0	0	0	0	0
P16039;F1NVA4	Nucleophosmin (NPM)	NPM1	2	2	2	6.1	6.1	6.1	32.632	0	3.7347	30081	1	0	1569.6	0	0	0	0	0	0	0	0
A0A1L1RJB3	Uncharacterized protei	MYOZ2	3	3	3	16.3	16.3	16.3	30.041	0	5.0064	31506	4	0	0	0	0	41019	0	0	0	0	0
A0A1L1RQQ0;A	Alpha-enolase;Alpha-e	ENO1;RCJMB04_2	22	15	13	55.1	43.3	36.9	47.332	0	182.49	2451200	83	284680	217060	249610	217210	170420	232430	350680	230640	346790	303050
Q5ZJL0;A0A1L1	Uncharacterized protei	NDUFS4;RCJMB04	2	2	2	13.4	13.4	13.4	21.32	0	3.8172	60679	4	0	0	0	0	0	15467	0	0	0	0
Q5ZMG9;A0A1	Uncharacterized protei	TCP1;TCP1 RCJMI	1	1	1	2.2	2.2	2.2	60.466	0	3.4969	16196	2	0	0	0	0	0	0	0	0	1217.9	0
A0A1L1RNL6	Uncharacterized protei	NACA	11	11	11	19	19	19	134.08	0	76.682	163600	23	0	11860	0	44839	0	0	0	28541	16775	0
P00508;F1P180;	Aspartate aminotransfe	GOT2	11	11	11	28.4	28.4	28.4	47.241	0	133.07	451450	42	34872	80953	116330	29077	11913	37252	33043	7956.4	19607	37995
F1NIJ6;Q5ZMU	Glucose-6-phosphate is	RCJMB04_1c14	20	20	20	42.3	42.3	42.3	59.226	0	256.18	2604200	142	258920	206080	381610	175450	140060	291180	336870	14007	203250	384880

P80566;A0A1L1	Superoxide dismutase [	SOD1	7	7	7	46.1	46.1	46.1	15.703	0	83.551	423270	35	0	34308	21195	62230	83543	48276	52036	91786	58106	36419
P24367;A0A1L1	Peptidyl-prolyl cis-tran	PPIB	2	2	2	14	14	14	22.413	0	3.9812	11505	3	0	4077.7	0	0	0	0	0	0	0	0
A0A1L1RUE3	Uncharacterized protei	HINTW	1	1	1	9.2	9.2	9.2	13.922	0.00278	3.0383	28619	1	0	0	0	0	0	0	0	0	0	12409
A0A1L1RX65;Q	Isocitrate dehydrogenas	IDH3A;RCJMB04_	2	2	2	5.7	5.7	5.7	39.79	0	3.2717	101850	3	0	0	0	0	0	0	0	0	0	17391
P28675;A0A1L1	Decorin (Bone proteog	DCN	4	4	4	18.8	18.8	18.8	39.686	0	16.317	79336	12	0	0	7879.1	0	0	0	0	15387	0	0
A0A1L1RXE9;D	Mitochondrial ubiquin	UQCRC2	6	6	6	18.1	18.1	18.1	45.772	0	34.287	280270	21	0	40635	53984	0	0	33661	46646	0	0	45445
D0VX32;A0A1L	Mitochondrial ubiquin	UQCRQ	1	1	1	19.8	19.8	19.8	9.4866	0	4.1561	6188.9	4	0	0	1818.4	0	0	0	0	0	0	0
Q5ZLC5;A0A1L	ATP synthase subunit b	ATP5F1B ATP5B R	16	16	16	42.4	42.4	42.4	56.627	0	175.86	1367300	102	186760	140070	184310	62986	75850	139450	215170	116760	79940	130390
O42283;A0A1L1	Heat shock protein 10;	HSPE1	7	7	7	65.7	65.7	65.7	11.083	0	27.354	612970	23	74815	63580	62345	98628	112950	53371	62956	144120	63402	22315
A0A1L1RZK1	Lactoylglutathione lyas	GLO1	11	11	11	58.9	58.9	58.9	20.553	0	44.471	816600	40	118950	95111	88393	94450	101170	88481	141980	0	101520	0
A0A1L1S091	Uncharacterized protein		11	11	11	67.6	67.6	67.6	20.729	0	172.45	774160	54	70267	90504	134000	143830	131450	87979	25198	169650	149880	63595
Q98TD1;A0A1L	PIT 54;Uncharacterize	PIT 54	4	4	4	10.9	10.9	10.9	50.821	0	36.459	55403	10	10096	10471	9583.1	0	8150.3	0	0	13263	0	0
F1NYA2;A0A1L	Uncharacterized protei	EIF4H;RCJMB04_1	5	5	5	28.3	28.3	28.3	27.635	0	42.457	218320	19	16254	19657	25877	32539	45850	20045	0	43009	0	22669
A0A3Q2UB77;A	Protein phosphatase 1 regulatory subunit 3A		5	5	5	5.1	5.1	5.1	117.24	0	11.669	49069	7	0	18287	0	0	0	0	0	0	0	0
A0A1L4FMK4	Toll-like receptor 7	TLR7	1	1	1	0.9	0.9	0.9	122.24	0.00277	3.0333	31014	4	0	0	0	0	0	0	0	0	0	8727.9
A0A1S5WGN8	Glyceraldehyde-3-phos	GAPDH	1	1	1	81.2	81.2	81.2	1.6118	0	3.7308	124190	8	0	0	0	0	0	0	0	0	0	21543
A0A3Q2TS15	Uncharacterized protein		4	4	4	0.6	0.6	0.6	673.18	0	7.109	49266	6	0	0	12543	0	0	0	0	0	0	10518
A0A3Q2UP73;A	Calcium voltage-gated channel auxiliary sub		2	2	2	5.6	5.6	5.6	42.862	0.01005	2.505	32815	2	0	0	0	2010	0	0	0	0	0	0
R4GI10;A0A3Q2	Peptide-methionine (R)	MSRB3	1	1	1	6	6	6	20.187	0	3.3798	6905.4	7	0	0	0	0	0	0	0	0	1145.8	0
A0A3Q2TUI0	Hes family bHLH transcription factor 2		1	1	1	16.2	16.2	16.2	10.936	0.00769	2.5668	184800	4	0	0	0	0	0	0	0	0	58753	0
A0A3Q2UBV6;F	Periostin;Uncharacteriz	POSTN	1	1	1	1.7	1.7	1.7	89.666	0	3.8685	22877	1	0	0	0	0	0	0	0	0	5013.3	0
A0A3Q2UIH0;A	Uncharacterized protei	CARHSP1	1	1	1	7.9	7.9	7.9	16.484	0	3.9292	6186.3	3	0	0	0	0	0	0	0	0	4394.8	0
A0A3Q2TXL9	p60=60 kDa stress-related protein (Fragmen		13	13	13	26.2	26.2	26.2	62.058	0	41.249	281410	39	22020	24748	21540	44706	46482	22827	32834	60582	36322	25990
A0A3Q2TYR0	Uncharacterized protei	MYOZ3	16	16	16	61.4	61.4	61.4	26.755	0	72.033	3225200	100	282400	290610	262170	490560	447700	317250	334080	503520	401420	335180
A0A3Q2TZD8;F	Uncharacterized protei	RAD23B	1	1	1	4.9	4.9	4.9	40.975	0	11.517	2418	4	0	0	0	0	0	621.5	0	0	0	0
Q1G1I6;A0A3Q2	PACSIN 3;Uncharacter	PACSIN3	2	2	2	6.2	6.2	6.2	50.742	0	6.3188	49393	8	0	0	0	0	0	0	0	0	12173	0
A0A3Q2U0S7;Q	DEAD-box helicase 3,X	DDX3X;DDX3X R	3	3	3	6.4	6.4	6.4	70.556	0	5.6755	54970	7	0	0	0	0	0	0	2484.6	0	0	0
A0A3Q2UJK8;A	Uncharacterized protei	VH1	2	2	2	23.8	23.8	23.8	10.531	0	3.483	58302	2	0	0	0	0	0	0	12376	0	0	0
A0A3Q2U3M8	Uncharacterized protein		8	8	8	49.7	49.7	49.7	21.572	0	57.714	201700	48	15328	6216.2	22758	25419	28699	19882	23610	28022	32109	20086
A0A3Q2U481	Uncharacterized protein		3	3	3	13	13	13	30.012	0	4.7213	184360	7	0	0	0	0	0	0	55688	0	0	0
A0A3Q2U4C6;Q	14-3-3 protein beta/alp	RCJMB04_3p21;SF	7	7	3	27.2	27.2	14.7	26.516	0	37.207	307200	24	45157	32347	35337	0	37330	24364	58589	36705	31506	0
P14732;A0A3Q2	Lamin B2;Lamin-B2	LMNB2	5	4	4	8.7	7.3	7.3	67.94	0	16.015	75477	11	0	6194.2	0	0	0	9895.3	12589	0	0	0
A0A3Q2UIK1;A	Glycogen [starch] synthase (EC 2.4.1.11)		2	2	2	2.1	2.1	2.1	97.893	0	4.0491	14461	4	0	0	0	0	0	0	0	2299.4	0	0
A0A3Q2U825	Uncharacterized protei	RPS28	3	3	3	46.4	46.4	46.4	7.8409	0	18.81	37536	11	9003.8	0	0	0	0	7652.6	6646.3	0	0	4870.7
Q5ZI86;F1P1B7;	Uncharacterized protei	NAP1L4;RCJMB04	1	1	1	3.5	3.5	3.5	42.831	0	5.0141	13148	4	0	0	0	0	7064.3	0	0	0	0	0
A0A3Q2U8Y0	Uncharacterized protei	STAC3	1	1	1	3.6	3.6	3.6	41.262	0.00275	2.9997	19521	2	0	0	0	0	0	0	0	0	5164.7	0
A0A3Q2UAA5	Uncharacterized protein		3	3	3	24.1	24.1	24.1	12.905	0	17.088	221200	19	23462	46307	7596.1	53385	0	0	9331.6	42149	20336	0
F6UW59;O9360	Apolipoprotein AIV;U	APOA4;apoAIV	2	2	2	7.4	7.4	7.4	40.682	0	4.0666	17571	4	0	0	0	0	0	0	0	0	0	3566.8
Q9PSW9;P0C1H	Histone H2B;Histone H	H2B-I;H2B-II;H2B-	7	7	7	57.1	57.1	57.1	13.964	0	36.393	1232000	38	130570	154230	180810	152890	196860	127890	41656	79395	145950	160420
P09860;A0A3Q2	Troponin C, slow skele	TNNC1	1	1	1	6.8	6.8	6.8	18.43	0	67.524	201610	8	0	0	0	0	0	0	0	18205	0	0
A0A3Q2UFS4	Myopalladin		3	3	3	6	6	6	67.909	0	14.568	8123.2	11	0	2475.3	0	0	0	0	0	0	0	0
A0A3Q2UHZ2	Uncharacterized protein		18	18	18	44.6	44.6	44.6	48.519	0	99.335	820530	51	48483	89732	88759	111950	75294	110010	90231	117740	116370	66396
F1N8U1;A0A3Q	Uncharacterized protei	MRPS36	1	1	1	11.2	11.2	11.2	11.645	0.00763	2.5467	0	1	0	0	0	0	0	0	0	0	0	0
A0A3Q3A4C6;A	Uncharacterized protein		76	7	5	34.1	3.6	2.5	302.27	0	25.056	167030	19	0	21414	33587	26003	33464	0	0	31262	0	0
A0A3Q3A6C1	Protein phosphatase 1 regulatory inhibitor su		3	3	3	33.3	33.3	33.3	9.9584	0	9.7777	19838	5	0	0	0	0	7560.9	0	0	0	0	0
A0A3Q3AC33	Alpha-1,4 glucan phosphorylase (EC 2.4.1.1		11	11	10	56.2	56.2	56.2	18.682	0	100.89	4838700	82	360890	565360	727130	217380	162850	598630	557620	142050	387620	919280
A0A3Q3AFR2	Uncharacterized protein		2	2	2	7	7	7	19.924	0	3.4744	28634	2	0	0	0	0	0	0	0	0	20548	0
A0A3Q3AHD7;C	MICOS complex subun	IMMT;RCJMB04_1	8	8	8	13.3	13.3	13.3	82.269	0	37.065	103040	20	22228	10699	10808	9839	0	23688	0	15401	16945	7930.9
A0A3Q3AHU9	Uncharacterized protei	LOC424401	7	7	7	52.2	52.2	52.2	13.06	0	19.338	379850	20	0	44901	0	77254	0	0	55002	83289	51487	39063
P80585;F1NVH2	Tubulin-specific chape	TBCA	1	1	1	14.8	14.8	14.8	12.679	0	8.5261	8780.4	4	0	0	0	0	0	0	3303.4	0	0	0
A0A3Q3AXT2	Uncharacterized protein		1	1	1	1.5	1.5	1.5	69.648	0	3.692	3385.3	1	0	0	0	0	0	0	0	4212.5	0	0
F1NUT9;A0A3Q	Troponin I1, slow skeletal type		9	9	9	48.1	48.1	48.1	20.879	0	51.057	309820	14	0	0	76447	0	349130	0	0	0	89743	0
A0A3Q8WI14	Glycerol-3-phosphate d	GPD1;GPD1L2	20	20	20	63.6	63.6	63.6	37.739	0	171.6	6646200	135	842550	732740	759430	542120	501370	707650	743690	622020	526070	868010
Q5ZME2;A0A45	Malate dehydrogenase,	MDH1 RCJMB04_2	7	7	7	30.5	30.5	30.5	36.543	0	40.608	121490	15	21964	19444	17543	0	0	13310	17026	0	0	16258
Q5ZK10;A0A45	NSFL1 cofactor p47;N	NSFL1C;NSFL1C R	3	3	3	8.9	8.9	8.9	40.64	0	6.0262	43360	8	0	0	0	13597	0	0	0	0	0	0
Q5ZM25;A0A45	Obg-like ATPase 1	OLA1;OLA1 RCJM	3	3	3	7.1	7.1	7.1	44.778	0	5.5483	22693	3	0	0	0	0	0	0	0	316.27	0	0
P08288;P08284;	Histone H1;Histone H1	HIST1H101;HIST1	2	2	2	6.8	6.8	6.8	21.803	0	4.3691	62206	1	0	0	0	0	0	0	0	0	15706	0
A0A452J889;P8	Prohibitin;Prohibitin tr	PHB	4	4	4	21	21	21	29.783	0	8.2814	15106	6	0	0	0	0	0	0	0	6213.1	0	0
P02467;A0A5H1	Collagen alpha-2(I) ch	COL1A2	12	12	12	10.8	10.8	10.8	128.99	0	177.06	2267900	65	86541	85549	0	911630	134300	155300	227820	885490	500990	195870
A0A860G8M5			7	7	2	11.4	11.4	3.3	83.212	0	17.798	489450	16	0	95983	0	100000	0	62802	73376	0	0	97894
P28497;A0M8U	F-actin-capping protein	CAPZA2	7	7	6	38.8	38.8	35.3	32.844	0	45.029	155960	29	23274	18855	0	18329	0	0	24190	13047	13738	0

P12902;A3RKL2	Nonhistone chromosom	HMGN5	1	1	1	12.4	12.4	12.4	11.357	0	3.417	1086.6	3	0	0	0	0	0	0	0	1020.4	0	0
A6BLM7	Titin isoform Ch12 (Fra	TTN	8	5	5	15	9.8	9.8	60.383	0	17.432	383390	19	59382	53274	57556	65588	65897	33054	0	43598	34847	43293
A6BLM8	Titin isoform b11 (Frag	TTN	7	1	1	9.3	1.2	1.2	110.95	0.00545	2.9389	14876	3	0	0	0	0	0	2891.6	0	0	0	0
A6BM71;Q98918	Connectin (Fragment);(	titin;TTN	79	79	73	11.1	11.1	10.1	904.79	0	323.31	6861400	353	780640	748080	808440	820840	902850	739310	688100	794010	688600	742280
Q8JIG5;A7UEB(	Alpha-1-acid glycoprot	OGCHI;ogchi OGCI	2	2	2	10.3	10.3	10.3	22.321	0	6.2631	26361	7	0	0	0	0	28729	0	0	0	0	0
Q5XKY5;Q5U78	5-aminoimidazole-4-ca	ATIC PURH;PURH	1	1	1	3.4	3.4	3.4	64.402	0	3.9426	888.56	1	0	0	0	0	0	0	0	1700.2	0	0
B5M200	Beta-actin (Fragment)	Bact	13	1	1	70.3	24.2	24.2	10.248	0.00779	2.6194	992.51	0	0	0	0	0	0	1032.3	0	0	0	0
D0EKR3;A0A3C	Peptidyl-prolyl cis-tran	PPIA	8	8	8	62.4	62.4	62.4	17.81	0	69.497	472840	45	37812	41828	42759	80437	89903	54878	27429	109220	79664	43267
D0VX28;F1NHV	Cytochrome b-c1 comp	UQCRH	2	2	2	37.7	37.7	37.7	9.04	0	22.578	88655	11	0	9455.4	0	0	8706.9	0	0	0	0	0
D0VX31;F1NAC	Mitochondrial ubiquinc	UQCRC1	3	3	3	8.3	8.3	8.3	49.441	0	19.101	100750	10	0	0	26862	0	0	0	13765	0	0	0
Q8UW59;D5M8	DJ-1 (Protein/nucleic a	DJ-1 PARK7;PARK	16	16	16	83.6	83.6	83.6	19.943	0	174.73	1447700	82	117090	159500	116070	182700	268950	107710	120410	349430	167160	127250
E1BQN0	NADH:ubiquinone oxidoreductase subunit S		2	2	2	18.4	18.4	18.4	22.395	0	17.712	0	2	0	0	0	0	0	0	0	0	0	0
Q683M9;Q5U7A	Adenylosuccinate lyase	ADSL;ADSL purB	3	3	3	6.8	6.8	6.8	54.502	0	7.1563	107000	9	18020	0	0	0	0	0	0	0	0	17412
E1BRT9	Uncharacterized protein	NDUFA5	4	4	4	50.9	50.9	50.9	13.448	0	15.023	71173	9	0	0	0	0	0	0	0	0	0	9360.2
E1BSH9	Uncharacterized protein	BHMT	9	9	9	30.6	30.6	30.6	45.01	0	34.754	694110	26	92143	74593	61801	0	0	76723	65638	126620	82982	87368
E1BSJ2	40S ribosomal protein S	RPS21	3	3	3	45.8	45.8	45.8	9.1183	0	4.2888	42928	3	0	0	0	0	19036	0	0	0	0	0
Q5ZME4;E1BSN	Alpha-1,4 glucan phosp	PYGB;RCJMB04_2	9	9	4	9.7	9.7	5.2	96.729	0	28.89	927450	43	76488	92312	119260	33938	0	130880	133490	0	48461	238570
E1BT93	Uncharacterized protein		4	4	4	7.5	7.5	7.5	56.79	0	7.7096	157940	9	32767	33991	0	0	0	26236	0	0	20465	0
E1BTT4	Hydroxyacyl-CoA dehydrogenase trifunction		2	2	2	5.3	5.3	5.3	50.849	0	3.3048	36086	4	0	0	0	0	0	7720.8	0	0	0	0
E1BTT8;P00340	L-lactate dehydrogenas	LDHA;LDHB	39	39	39	94.6	94.6	94.6	36.502	0	323.31	22261000	347	2387900	2520000	2943000	1504100	1E+06	2740500	2620200	405480	1700000	2828000
E1BU93	Myozenin 1		18	18	18	67.6	67.6	67.6	30.905	0	229.88	5404500	165	441390	461260	408230	840160	675060	570720	464410	945140	700450	506200
P68246;E1BUD3	Troponin I, fast skeletal	TNNI2	27	27	27	77.6	77.6	77.6	21.234	0	323.31	44415000	497	3912200	4606600	4497600	6854500	1E+07	3656100	3568600	7939800	5609700	3590200
E1BVT3	Malate dehydrogenase (	MDH2	19	19	19	66.2	66.2	66.2	35.655	0	220.39	1729500	144	192440	201940	240670	131400	139360	140980	198080	199940	143850	166940
E1BVU4	SH3 domain-binding glutamic acid-rich-like		2	2	2	26.8	26.8	26.8	12.557	0	22.16	9968.6	6	0	1373.6	3565.1	0	0	0	0	0	0	0
E1BWS9	Uncharacterized protein	HYPK	1	1	1	8.5	8.5	8.5	14.111	0.00771	2.5702	13806	1	0	0	0	0	0	0	7576.2	0	0	0
E1BX17;A2NBE	Uncharacterized protein	MYLK2	8	8	8	14.3	14.3	14.3	87.197	0	34.935	402610	15	38816	0	32101	42690	70657	0	64163	56948	0	48760
E1BXC2	S-formylglutathione hy	ESD	2	2	2	7.4	7.4	7.4	31.559	0	4.2906	46672	3	0	0	11004	0	0	0	0	0	0	0
E1BXG9	Uncharacterized protein	PPID	3	3	3	10.3	10.3	10.3	40.478	0	4.6846	31484	7	0	0	0	0	0	0	0	0	7072.8	0
E1BXI3	Uncharacterized protein	PDCD5	6	6	6	47.6	47.6	47.6	14.219	0	24.281	40938	16	0	0	0	0	6801.7	0	0	0	2826.5	0
E1BZ03	Adenylyl cyclase-assoc	CAP2	2	2	2	5.4	5.4	5.4	53.197	0	8.8182	17043	6	0	0	0	0	0	0	0	0	0	5425.6
E1C043	Cytochrome c oxidase subunit 5A		3	3	3	22.9	22.9	22.9	15.968	0	28.253	71369	19	0	9969	8886.6	11789	14517	9321.9	0	11814	8475.8	0
E1C0Q5	Uncharacterized protein	ACAT1	3	3	3	10.7	10.7	10.7	44.09	0	15.875	65044	14	0	0	0	0	0	0	0	0	1452.6	0
E1C4V1	ATP synthase-coupling	ATP5PF	8	8	8	57	57	57	12.36	0	49.87	648010	53	62049	55639	66745	105900	188500	62097	32048	122970	72494	58027
E1C516	Pyridoxal phosphate ho	PROSC PLPBP	2	2	2	9.2	9.2	9.2	29.469	0	9.7113	5623.6	5	0	0	0	0	6150.1	0	0	0	0	0
E1C5K0	Uncharacterized protein	HOMER3	1	1	1	5.9	5.9	5.9	40.017	0	5.5639	1781.5	1	0	0	0	0	0	0	0	1060.3	0	0
E1C658	ATP synthase subunit d	ATP5PD	11	11	11	58.4	58.4	58.4	18.343	0	133.67	940320	55	119330	103330	92427	143930	186270	89076	93401	187210	109530	68228
E1C6C9	Uncharacterized protein	NDUFB10	3	3	3	13.1	13.1	13.1	20.497	0	9.6917	35894	5	0	0	0	0	0	0	0	16818	0	0
E1C6N5	Acetyltransferase comp	DLAT	4	4	4	7	7	7	66.522	0	5.9432	61508	5	0	0	0	0	0	0	0	22893	0	0
E1C6V0	Uncharacterized protein	HSPB8	2	2	2	13.2	13.2	13.2	21.734	0	6.3808	32739	4	0	0	0	0	0	0	0	0	9920.3	0
Q5ZMN3;F1N83	Prohibitin-2	PHB2;PHB2 RCJMI	1	1	1	4	4	4	33.336	0.00775	2.5818	1844.7	1	0	0	0	0	0	0	1588	0	0	0
F1N9H4;A0A1I7	Elongation factor 1 alpl	EEF1A;EEF1A1;EE	10	10	10	26.1	26.1	26.1	50.498	0	110.32	2071900	55	176540	205870	249320	279730	77855	265810	262250	200090	201990	220370
Q98TF8;F1N9J4	60S ribosomal protein L	RPL22	1	1	1	10.2	10.2	10.2	14.729	0	3.5502	2389.7	1	0	0	0	0	4642.7	0	0	0	0	0
Q92108;Q6QAZ	Annexin;Annexin A1 (A	ANXA1;ANXA1 AI	1	1	1	10.8	10.8	10.8	14.398	0.00542	2.9193	2293.2	3	0	0	0	0	0	0	0	0	0	573.8
Q5F3M8;F1N9Z	Succinyl-CoA:3-ketoac	OXCT1;RCJMB04_	1	1	1	3.5	3.5	3.5	56.185	0	3.6652	666.97	2	0	0	0	0	0	0	0	0	0	666.5
P11009;F1NAD3	Creatine kinase S-type,	CKMT2	18	17	17	55.1	52.5	52.5	47.083	0	185.51	269210	57	35777	38302	42091	19344	24037	26228	19140	11156	27155	27091
Q5F4B1;F1NAX	Glycerol-3-phosphate p	PGP;PGP RCJMB0	4	4	4	17.3	17.3	17.3	32.995	0	12.679	100480	8	0	0	0	0	0	0	0	0	38075	0
F1NBV0;Q5ZJF	Peroxiredoxin-6;Peroxi	PRDX6;PRDX6 RC	17	17	17	67.4	67.4	67.4	25.076	0	116.69	1065900	99	95954	122890	72147	163240	140990	101840	102090	198060	119010	101480
F1NCA2	Glycerol-3-phosphate d	GPD2	7	7	7	11.6	11.6	11.6	80.751	0	21.691	62659	13	21049	10088	0	0	0	0	0	0	0	0
R4GFD0;F1ND0	Uncharacterized protein	PSME4	1	1	1	0.4	0.4	0.4	199.08	0.00531	2.8191	1148000	11	0	0	0	0	0	0	0	0	0	111090
F1NGM2	Uncharacterized protein	COQ8B	2	2	2	3.7	3.7	3.7	72.489	0	4.7457	79823	8	0	0	0	0	0	0	0	0	0	12632
Q5ZLF0;F1NH2	Hsc70-interacting prote	ST13;ST13 FAM10	3	3	3	11.6	11.6	11.6	40.158	0	6.179	27608	3	0	0	6554.6	0	0	0	0	0	0	0
F1NH40	Uncharacterized protein	SYNPO2	13	13	13	11.2	11.2	11.2	137.54	0	69.095	760850	47	78285	27781	63002	126150	121230	101420	105600	136120	122030	79273
F1NH93	Uncharacterized protein	RPS20	2	2	2	16	16	16	13.374	0.00789	2.7011	0	2	0	0	0	0	0	0	0	0	0	0
F1NHA9;Q9PU4	PDZ and LIM domain p	PDLIM3;PDLIM3 A	15	15	15	57	57	57	39.565	0	158.9	876820	77	100080	69291	80146	109280	100670	57379	109540	119910	164890	87036
F1NHH1	Uncharacterized protein	CSTB	3	3	3	45.9	45.9	45.9	11.159	0	5.5179	18393	2	0	0	0	0	0	0	0	19522	0	0
Q6EE33;Q5ZK8	S-(hydroxymethyl)gluta	ADH5;RCJMB04_1	1	1	1	3.5	3.5	3.5	39.124	0.01003	2.4957	7191.5	1	0	0	0	0	0	0	0	0	11419	0
F1NIC5	Uncharacterized protein	TIMM8A	2	2	2	20.2	20.2	20.2	10.686	0	5.4029	29497	4	0	0	0	0	23461	0	0	0	0	0
F1NIP5;A0A3Q	Ribose-phosphate pyro	PRPS1;PRPS2 RCJ	3	3	3	14.8	14.8	14.8	34.747	0	7.2253	3993.1	3	0	0	0	1103.7	0	0	0	0	0	0
F1NJ08;A0A1L1	Internexin neuronal int	GFAP;KRT5;KRT6	39	39	34	74.1	74.1	67.2	53.215	0	250.73	4483800	168	562700	377210	543320	360120	393340	589540	406740	887130	441150	678550
Q90662;F1NJM8	Synemin;Uncharacteriz	SYNM	8	8	8	6	6	6	182.19	0	33.328	116130	16	0	14187	19952	0	23122	10297	0	24855	0	16932
Q5F3N1;F1NJV	Protein-L-isoaspartate (	PCMT1;PCMT1 RC	8	8	8	36.4	36.4	36.4	24.691	0	38.813	505860	23	40281	44049	49479	103490	79920	37206	59626	147440	52115	28964

F1NK75;A0A1D	Uncharacterized protein	TPM4	24	3	3	42.6	10.9	10.9	32.785	0	21.504	255170	34	0	30534	32330	17303	28342	28374	33643	30208	26524	29092
Q5F472;F1NK96	Uncharacterized protein	PDIA6;RCJMB04_2	1	1	1	5.7	5.7	5.7	26.885	0	3.3289	11074	7	0	0	0	0	0	0	1975	0	2627.8	1766.9
F1NM49;Q9IBD	Myosin heavy chain;My	MYH15	23	3	3	9	1.7	1.7	223.6	0	70.97	135390	9	0	0	0	0	0	0	46685	0	0	0
F1NN16	40S ribosomal protein S	RPS7	2	2	2	18	18	18	22.112	0	5.4145	6376.2	7	0	0	0	0	0	0	0	0	1455.4	0
F1NN63;Q2UZR	Phosphoglucomutase 1;	PGM1;PGM5	42	42	42	70.8	70.8	70.8	61.548	0	323.31	8472000	256	1268100	912820	1054200	619310	429230	1030500	965300	331860	666380	1205200
F1NNW0;Q0217	M-protein, striated mus	MYOM2	49	49	49	40.8	40.8	40.8	163.41	0	323.31	4047700	174	323940	340300	514620	267160	257620	450250	627750	377220	436350	497830
F1NPL9	Uncharacterized protein	COX17	1	1	1	16.4	16.4	16.4	7.2084	0.00536	2.8714	0	1	0	0	0	0	0	0	0	0	0	0
Q9PU45;F1NQD	Radixin	RDX	4	4	4	6.9	6.9	6.9	68.554	0	8.4812	159250	7	0	31241	0	32396	0	0	0	0	37922	0
F1NQW8;Q5ZM	Serine/arginine-rich spl	SRSF1;SRSF1 SFRS	2	2	2	7.7	7.7	7.7	27.791	0	3.4903	0	3	0	0	0	0	0	0	0	0	0	0
F1NRM8	Endoplasmic reticulum	ERP29;ERP29 RCJI	3	3	3	9.5	9.5	9.5	28.191	0	3.9963	9758.1	4	0	0	0	0	0	0	0	4290.1	1878	0
F1NSZ4	Uncharacterized protein	NPEPL1	2	2	2	7.6	7.6	7.6	56.089	0	3.6537	2436.7	2	0	1052.2	0	0	0	0	0	0	0	0
F1NT19;Q9DDJ	Superoxide dismutase (	MNSOD	2	2	2	10.3	10.3	10.3	24.859	0	12.509	17849	5	2429.8	0	0	0	0	0	0	0	0	0
F1NT28	Uncharacterized protein	PPA1	3	3	3	12.1	12.1	12.1	32.637	0	6.4124	10985	3	0	0	0	0	0	0	0	0	3524.3	0
P00504;F1NTM7	Aspartate aminotransfer	GOT1	10	10	10	28.2	28.2	28.2	45.935	0	78.89	638760	49	101020	67040	78040	34805	37022	64894	53922	60041	87665	83722
F1NU17;P51903	Phosphoglycerate kinas	PGK	47	47	47	83.2	83.2	83.2	44.666	0	323.31	60616000	715	6083900	6460200	6608000	6380600	5E+06	6774000	6005400	5678700	5928400	6276600
F1NU56	Uncharacterized protein	RPS25	2	2	2	15.2	15.2	15.2	13.712	0	3.4786	0	3	0	0	0	0	0	0	0	0	0	0
Q6DRR5;F1NU(	Fatty acid binding prote	FABP;FABP7;PMP	6	5	5	51.1	45.9	45.9	14.816	0	13.799	382070	27	44198	26065	0	0	0	46257	38178	91166	98409	37416
F1NVD4	Adenylosuccinate synth	ADSSL1 ADSS1	8	8	8	26.5	26.5	26.5	50.097	0	52.436	107540	19	0	10921	12697	13524	0	12222	15515	0	15329	0
Q9W6F5;F1NVF	GC, vitamin D binding	VTDB	2	2	2	6	6	6	53.686	0	5.0473	31275	4	0	0	0	0	0	0	0	0	0	2611.6
Q5ZJ57;F1NXN	Uncharacterized protein	NDUFS1;RCJMB04	1	1	1	1.4	1.4	1.4	79.576	0.00528	2.7448	29428	3	0	0	0	0	0	0	0	0	0	6864.8
P70079;F1NXR0	Creatine kinase U-type,	CKMT1;CKMT1A	2	1	1	8.6	6	6	47.103	0.00273	2.9551	2648.7	0	0	0	0	0	0	0	0	0	0	1693
F1NY09	Uncharacterized protein	C1H11ORF54	4	4	4	18	18	18	34.679	0	11.99	14641	21	3641.9	0	0	4705.4	0	0	0	0	0	0
F1NY41;A0A1L	Uncharacterized protein	BAG3;RCJMB04_2	7	7	7	19.6	19.6	19.6	61.178	0	23.712	272720	20	0	30450	21481	38313	55011	12052	24692	51615	37968	40828
F1NYA9;Q9YG(	Elongation factor 1-beta	EEF1B;EEF1B2;RC	2	2	2	7.1	7.1	7.1	24.718	0.00773	2.5776	73240	3	0	0	0	0	0	0	0	19139	0	0
F1NYB1;P09206	Tubulin beta chain;Tub	TUBB2A;TUBB4B	8	8	8	18	18	18	49.86	0	51.056	139830	18	17240	17643	22160	9190.3	0	14216	19590	0	0	20308
F1NYI3	tRNA-splicing ligase R	RTCB	2	2	2	5.1	5.1	5.1	55.242	0	5.0913	59063	3	0	0	0	13974	0	0	0	0	0	0
F1NZ04;Q5ZLU	Amphiphysin;Bridging	AMPH;BIN1;BIN2	12	12	12	34.6	34.6	34.6	48.701	0	124.92	426240	49	40635	31096	31779	40445	58769	37813	48268	62206	67230	54691
Q5ZM98;F1NZ8	Stress-70 protein, mito	HSPA9;HSPA9 RCJ	14	14	14	22.4	22.4	22.4	73.191	0	79.678	316680	36	34390	36917	33322	46032	42670	35239	27733	60556	36167	27698
F1P0N2	AMP deaminase (EC 3.5.4.6)		6	1	1	10.5	2.3	2.3	81.333	0	22.442	3656.8	3	0	0	0	0	0	1481.2	0	0	0	0
F1P241	CRK like proto-oncogene, adaptor protein		1	1	1	6.2	6.2	6.2	21.148	0.00767	2.5605	4752.3	3	0	3802.5	0	0	0	0	0	0	0	0
F1P2A1	Uncharacterized protein	ATOX1	1	1	1	25.4	25.4	25.4	7.984	0	3.9296	63127	4	0	0	0	0	0	0	0	21194	0	0
F1P2E9	Myopalladin		2	2	2	2.6	2.6	2.6	109.52	0	3.3209	19046	2	0	0	0	0	0	0	0	0	0	5492.5
P14448-2;P1444	Fibrinogen alpha chain;	FGA	1	1	1	3.7	3.7	3.7	56.173	0	5.0298	1058.1	1	0	0	0	0	0	0	910.79	0	0	0
F1P593;Q00649;	Heat shock protein beta	HSPB1	17	17	17	72.7	72.7	72.7	21.826	0	253	4132700	252	304240	369030	333050	606500	984550	409560	325740	748310	620580	299630
Q5ZJ45;F1P5T2	Phosphorylase kinase c	RCJMB04_20n15	2	2	2	6.7	6.7	6.7	45.395	0	4.9016	2706.1	2	0	2165.2	0	0	0	0	0	0	0	0
F1P5V6;P02606	Myosin light chain 1, c	MYL3	16	14	13	75.8	69.6	69.6	21.911	0	74.227	1268700	50	6756.1	0	126700	0	2E+06	0	7511.3	0	131150	0
Q9DEA4;Q9PU(	Skeletal muscle type tro	E-Tmod;skTmod;Sk	11	11	11	33.6	33.6	33.6	37.276	0	90.865	1684800	49	217190	195000	166670	158710	170610	206990	215740	260980	184710	141550
H9L3K0;H9L07	Tropomyosin 3 alpha is	TPM3;TPM3 RCJM	28	2	2	41.9	4.2	4.2	32.818	0	9.4474	10884000	25	0	1270700	0	753090	0	0	0	0	722500	0
O57535	Nucleoside diphosphate	NME1;NME3	10	10	10	66.7	66.7	66.7	17.288	0	121.52	823250	55	104300	89141	80118	141150	117070	60189	80683	87975	77234	91641
Q7ZZH5;P12620	Fast skeletal muscle tro	TNNT3	37	37	7	55.4	55.4	8.4	29.676	0	323.31	53379000	913	4445400	4822200	4935300	8648800	8E+06	6058700	5499300	6284100	7183300	5905800
P62149;O93410;	Calmodulin;Calmodulin	CALM CAM RCJM	6	5	4	44.3	39.6	26.2	16.837	0	42.554	149380	13	8579.8	0	0	0	22800	0	16936	0	0	45108
P00356	Glyceraldehyde-3-phos	GAPDH;GAPDH G	45	45	44	84.1	84.1	84.1	35.704	0	323.31	1.07E+08	934	9859900	9826000	1.2E+07	9983100	6E+06	10595000	11549000	9136100	10216000	1.2E+07
P00548;A0A1D5	Pyruvate kinase (EC 2.	PKM	56	56	56	91.1	91.1	91.1	58.014	0	323.31	74984000	1001	8842100	7425500	9822700	5767000	4E+06	8129900	9378500	4997200	5762500	8894100
P00565	Creatine kinase M-type	CKM	52	52	50	83.7	83.7	83.7	43.328	0	323.31	70907000	1538	5756200	6851500	6153800	1.1E+07	8E+06	5711400	6502800	5355000	8484200	7161200
P00940	Triosephosphate isomer	TPI1	34	34	34	93.5	93.5	93.5	26.62	0	323.31	1.37E+08	1285	12002000	11678000	1.2E+07	2.1E+07	2E+07	14381000	14678000	20287000	19682000	1.4E+07
P01994	Hemoglobin subunit alp	HBAA	6	5	5	42.3	37.3	37.3	15.429	0	122.7	619770	53	127280	85020	56496	66714	52063	91317	54452	76089	57788	65351
P02001	Hemoglobin subunit alp	HBAD	10	10	9	63.8	63.8	58.9	15.695	0	59.641	1520600	42	265840	191250	118930	176730	152350	180910	0	451490	138080	149830
P02112;Q90864	Beta-globin protein (Fr	HBB;HBE;HBE1	6	6	6	48.3	48.3	48.3	16.466	0	31.83	411150	29	0	60341	45749	47670	0	46980	58254	0	57258	45642
P02197	Myoglobin	MB	8	8	8	67.5	67.5	67.5	17.422	0	33	155840	12	0	0	67526	0	89656	0	0	0	43777	0
Q92069;P70082;	Histone H2A;Histone H	H2A2BL;H2AFV H	5	5	5	35.7	35.7	35.7	13.94	0	55.719	1065000	48	71220	114770	66877	157290	120330	130240	133810	223590	141220	109700
P02542;O73665	Desmin;Desmin (Fragm	DES	26	22	21	54.1	48.7	47	53.512	0	226.19	2177900	95	292340	290090	210250	199600	189990	173040	251560	227000	180440	286980
P02588	Troponin C, skeletal mu	TNNC2	12	12	11	73.6	73.6	69.3	18.375	0	113.25	2999600	98	275010	236430	367590	275240	390650	239520	221680	113790	132130	480330
P02604	Myosin light chain 1, skeletal muscle isoform		25	25	5	89.6	89.6	24.5	20.899	0	323.31	96560000	814	10787000	9288700	8841600	9354300	2E+07	10442000	8127400	9259900	8623100	1.2E+07
P02605	Myosin light chain 3, skeletal muscle isoform		23	3	3	96	16	16	16.71	0	129.16	1665800	47	157450	176370	203900	175330	303200	133720	169600	0	183630	188130
P02609	Myosin regulatory light	MYLPF	24	24	24	95.8	95.8	95.8	18.839	0	323.31	47255000	349	4131200	5109200	7278400	2226400	2E+06	5920700	3629700	1373400	1487000	9278600
P02789;E1BQC2	Ovotransferrin;Ovotran	TFEW	7	7	7	10.2	10.2	10.2	77.776	0	35.259	108240	20	0	14588	12944	18932	0	0	17705	22728	18538	18592
P05081	Adenylate kinase isoen	AK1	35	35	35	92.8	92.8	92.8	21.683	0	323.31	22570000	520	2061100	1844900	1801600	3165700	3E+06	2126800	1730700	3993800	3021300	2343800
P07032	Acylphosphatase-1 (EC	ACYP1	4	4	4	45.5	45.5	45.5	11.15	0	42.112	364910	9	0	57258	0	32744	39496	48433	0	0	41046	0
P07322	Beta-enolase (EC 4.2.1	ENO3	43	43	35	69.1	69.1	55.8	47.196	0	323.31	1.03E+08	1293	12561000	11291000	1.2E+07	1.1E+07	9E+06	9757800	9474200	9225500	10442000	1.2E+07
P07341;B4Z858	Fructose-bisphosphate	ALDOB	6	4	4	15.9	12.1	12.1	39.295	0	9.5571	328900	20	59035	0	0	0	0	0	0	186250	0	0
P07583	Beta-galactoside-bindir	CG-1B	2	2	2	20.7	20.7	20.7	15.063	0	4.5708	16303	3	0	0	0	0	0	0	0	9809.9	0	0

P07630	Carbonic anhydrase 2 (	CA2	6	6	6	23.5	23.5	23.5	29.007	0	67.705	81892	13	25464	15235	0	0	0	0	0	0	0	0
P08250	Apolipoprotein A-I (Ap	APOA1	17	17	17	57.2	57.2	57.2	30.68	0	91.726	2377400	109	229930	301820	157240	374220	435890	240080	261760	271940	381210	229800
P08629	Thioredoxin (Trx)	TXN	1	1	1	14.3	14.3	14.3	11.7	0.00538	2.8949	35921	2	0	0	0	0	0	0	0	1757	0	0
P0CB50	Peroxiredoxin-1 (EC 1.	PRDX1;PRDX4	6	6	6	33.2	33.2	33.2	22.314	0	101.6	616190	18	0	75649	64932	69259	79048	66739	104880	129450	76396	0
P12003;P12003-1	Vinculin (Fragment);Vi	VCL VINC1;vinculi	3	3	3	3	3	3	124.56	0	4.0602	152910	2	0	0	0	0	0	68327	0	0	0	0
P12620-4;P1262(	Troponin T, fast skeleta	TNNT3	34	4	4	55	8	8	29.648	0	8.5968	604630	14	0	71476	108200	0	206310	46174	50518	0	89352	0
Q5ZLP3;P13127	F-actin-capping protein	CAPZA1;RCJMB04	3	2	2	18.1	13.1	13.1	22.955	0	6.6651	10404	5	0	0	0	0	0	0	7830.7	0	0	0
P13538	Myosin heavy chain, skeletal muscle, adult		191	191	0	67.9	67.9	0	223.14	0	323.31	1.36E+08	1965	11648000	14068000	1.1E+07	1.7E+07	2E+07	16212000	15395000	13738000	17307000	1.2E+07
P13648	Lamin A/C;Lamin-A	LMNA	13	13	12	23	23	21.8	73.164	0	39.726	342270	34	30010	35934	37693	39352	44632	42824	33463	61017	45764	33437
P14315;P14315-2	F-actin-capping protein	CAPZB	7	7	7	28.2	28.2	28.2	31.364	0	26.203	547470	18	60568	53161	73438	50084	30420	67442	84703	72538	0	62410
P15988-2;P1598	Collagen alpha-2(VI) c	COL6A2	2	2	2	2.5	2.5	2.5	97.62	0.00275	2.9915	31088	2	0	0	0	0	0	5532.4	0	0	0	0
P16419	Myosin binding protein	MYBPC2;MYBPC3	65	65	63	54.5	54.5	53.1	126.94	0	323.31	18032000	508	1593600	1731300	2606800	1464300	1E+06	2055800	2394100	2063600	1918300	1965700
P19121	Serum albumin (Alpha-	ALB	33	1	1	53.3	2.1	2.1	69.918	0	7.9676	33645	11	3280.6	0	0	5316.4	8275.9	0	2906.2	0	0	2665.9
P19352;Q05706;	Beta tropomyosin (Frag	BRT-1 TPM2;BRT-	35	20	12	72.5	48.2	23.6	32.776	0	323.31	2540800	85	27224	23570	986900	89531	670280	164120	132850	273600	225210	290510
P20111;P20111-2	Actinin alpha 4;Alpha-a	ACTN1;ACTN2;AC	53	53	45	60.8	60.8	54.1	104.27	0	323.31	3646200	246	344080	316440	414260	109660	43787	424220	803740	162130	298290	461710
P20763;R9PXM5	Ig lambda chain C region;Uncharacterized p		2	2	2	19.4	19.4	19.4	11.361	0	12.423	20823	8	0	0	0	0	0	0	0	0	0	2346
P21566;A0A452	Cofilin-2;Cofilin-2 (Co	CFL2	12	12	12	75.3	75.3	75.3	18.661	0	66.754	360460	59	29305	29401	31941	42764	49765	32202	28247	109290	46500	34011
Q6L755;Q6L754	Beta-2-microglobulin;E	B2M;B-F-S-b2m01;	3	3	3	28.6	28.6	28.6	13.085	0	6.7812	61960	11	0	0	0	0	17807	0	0	0	0	17356
P23614	Brain acid soluble prote	BASP1	1	1	1	5.7	5.7	5.7	25.438	0	5.7533	0	1	0	0	0	0	0	0	0	0	0	0
P24479	Protein S100-A11 (Cal	S100A11	1	1	1	10.9	10.9	10.9	11.413	0.00541	2.9036	2153.5	2	0	0	0	0	0	0	0	985.03	0	0
P34065	Proteasome subunit bet	PSMB5	1	1	1	6.2	6.2	6.2	27.045	0.00759	2.528	0	1	0	0	0	0	0	0	0	0	0	0
P50890	40S ribosomal protein S	RPSA LAMR1	1	1	1	4.4	4.4	4.4	33.021	0	3.3556	0	1	0	0	0	0	0	0	0	0	0	0
P51890	Lumican (Keratan sulfa	LUM LDC	7	7	7	21.6	21.6	21.6	38.642	0	20.982	760930	32	0	79387	66862	62614	85008	104370	110090	132960	160470	157050
P51901	Annexin A6 (67 kDa ca	ANXA6 ANX6	8	8	1	12.7	12.7	1.5	75.218	0	21.915	93250	22	9374.1	14458	17789	0	0	11889	15960	0	0	11285
P62801;P70081;(	Histone H4;Histone H4	H4-I;H4-II;H4-III;H	6	6	6	55.3	55.3	55.3	11.367	0	20.734	258100	29	0	0	19849	25210	20703	28073	39752	134870	32086	35565
P67881	Cytochrome c	CYC	6	6	6	49.5	49.5	49.5	11.71	0	12.987	143710	20	0	0	22851	0	0	20311	30718	27590	20161	12588
P68139;P68034;I	Actin, alpha cardiac mu	ACT85CL;ACTA1;A	37	37	15	80.4	80.4	48.5	42.051	0	323.31	1.5E+08	768	20521000	17716000	1.6E+07	1E+07	8E+06	17891000	20528000	15439000	14879000	1.8E+07
P68497	Metallothionein (MT)		2	2	2	42.9	42.9	42.9	6.4616	0	4.6144	2903	2	0	1628.8	0	0	0	0	0	0	0	0
P79757;A0A3Q2	Connectin/titin (Fragment);Uncharacterized		19	19	19	19.1	19.1	19.1	113.9	0	104.76	1242000	57	146990	130330	139440	153880	179470	122650	120550	162980	100330	157070
Q02960	Macrophage migration	MIF	1	1	1	14.8	14.8	14.8	12.484	0	3.1591	7478.2	8	0	0	0	0	0	0	710.08	4632.8	0	1137.3
Q05623;E1C597	Myosin-binding protein	MYBPH	28	28	28	54	54	54	58.678	0	323.31	5188100	155	608800	694400	583200	337820	226440	524660	692680	517900	421860	689350
Q09121	Eukaryotic translation i	EIF5A1 RCJMB04_	5	5	3	28.5	28.5	17.4	15.889	0	20.87	405620	21	0	53309	53773	40566	40325	47697	45394	0	32722	67507
Q58I02;K9J9Q6	Nicotinamide phosphor	PBEF1 NAMPT	2	2	2	5.5	5.5	5.5	55.586	0	5.0243	68114	5	0	0	0	0	0	0	0	0	14469	0
Q5F3D2	Uncharacterized protein	HNRNPH3 RCJMB	3	3	3	9.6	9.6	9.6	36.656	0	7.7662	107060	3	0	0	0	0	0	0	0	0	0	15200
Q5F3Z3;Q90879	Ubiquitin conjugating e	UBE2V1;UBE2V2 I	3	3	1	18.1	18.1	6.2	16.305	0	6.435	153860	8	0	0	0	45308	0	0	0	49773	0	0
Q5F424	Uncharacterized protein	CCT2 RCJMB04_3r	6	6	6	13.8	13.8	13.8	57.431	0	13.882	41757	12	0	0	0	0	0	0	0	0	3448.8	0
Q5ZHP2	Uncharacterized protein	OARD1 RCJMB04_	4	4	4	21.2	21.2	21.2	20.906	0	8.9628	49977	9	0	0	0	0	0	0	9491.9	0	0	8756.7
Q5ZHV4	Phosphoglycerate muta	BPGM RCJMB04_3	9	9	9	33.2	33.2	33.2	29.736	0	34.012	393350	25	57894	38098	32105	40026	40021	36711	55023	43223	38249	33584
Q5ZHW8	Uncharacterized protein	RPS14 RCJMB04_3	1	1	1	8.6	8.6	8.6	16.273	0	5.2112	10205	2	0	0	0	0	0	0	0	0	0	2338.7
Q5ZIR7	Uncharacterized protein	RCJMB04_23p16	1	1	1	6	6	6	15.079	0.00781	2.6274	14192	9	0	0	0	0	0	0	0	0	0	1612.7
Q5ZIT7	Uncharacterized protein	PSMC3 RCJMB04_	1	1	1	4	4	4	47.198	0.00532	2.8252	2696.6	2	0	0	0	0	0	0	0	0	2175.2	0
Q5ZJ54	T-complex protein 1 su	CCT6 RCJMB04_2(	2	2	2	3.6	3.6	3.6	57.644	0	3.4491	15753	4	0	0	0	0	0	0	0	0	0	1497.4
Q5ZJA7	Uncharacterized protein	OGDH RCJMB04_1	1	1	1	1.2	1.2	1.2	115.18	0	3.178	4547.1	3	0	0	0	0	0	0	2131.8	0	0	0
Q5ZJL3	Uncharacterized protein	PSMF1 RCJMB04_	2	2	2	9.6	9.6	9.6	29.142	0	4.1793	0	2	0	0	0	0	0	0	0	0	0	0
Q5ZJX9	Proteasome subunit alp	PSMA5 RCJMB04_	1	1	1	7.9	7.9	7.9	26.447	0	4.3673	18745	2	0	0	0	0	0	0	0	0	0	4599.7
Q5ZJY1	6-phosphogluconolacto	PGLS RCJMB04_14	3	3	3	18.4	18.4	18.4	26.213	0	7.3234	14185	11	0	0	0	0	3724.3	0	0	0	0	0
Q5ZK23	Uncharacterized protein	GLRX5 RCJMB04_	2	2	2	18.5	18.5	18.5	17.425	0	4.5835	7918.6	2	0	0	0	0	0	0	0	15151	0	0
Q5ZKA4	Eukaryotic translation i	EIF3J EIF3S1 RCJM	1	1	1	5.2	5.2	5.2	28.724	0	4.3656	13326	2	0	0	0	0	0	0	0	0	0	132.42
Q5ZKE7	UMP-CMP kinase (EC	CMPK CMPK1 RCJ	2	2	2	14.8	14.8	14.8	22.172	0	4.5082	18360	4	0	0	0	0	0	3689	0	6737	0	0
Q5ZL72	60 kDa heat shock prot	HSPD1 HSP60 RCJ	18	18	18	39.6	39.6	39.6	60.972	0	137.47	694150	82	65600	73121	69305	65586	120740	59480	64675	182910	65523	35273
Q5ZL80	Uncharacterized protein	HNRNPM RCJMB0	2	2	2	4.4	4.4	4.4	76.022	0	16.077	40786	5	0	0	0	9151.2	0	0	0	0	0	0
Q5ZLD1	Uncharacterized protein	FH RCJMB04_6k20	3	3	3	6.1	6.1	6.1	54.299	0	13.424	90754	3	0	37510	0	0	0	13714	0	0	0	0
Q5ZLN1	Phosphoglycerate muta	PGAM1 RCJMB04_	34	34	34	96.9	96.9	96.9	28.898	0	323.31	17340000	461	1875900	1719200	1575300	1682600	1E+06	1649400	2091200	1489900	1818200	1669900
Q5ZLR5	Cytochrome b-c1 comp	UQCRFS1 RCJMB(	3	3	3	15.4	15.4	15.4	29.386	0	11.759	18652	14	0	0	0	0	0	0	0	4889	0	0
Q5ZM32	Dihydrolipoyl dehydrog	DLD RCJMB04_3f8	6	6	6	10.6	10.6	10.6	53.974	0	15.912	211160	18	61034	12762	0	19383	0	19119	0	0	27964	22892
Q5ZM94;F1NT9	Uncharacterized protein	C1H21ORF33;RCJM	5	4	4	24.7	21.6	21.6	27.371	0	26.071	188570	17	30425	0	27149	30177	27444	22048	20601	28431	15443	0
Q5ZMC0;A0A3(	Endothelial differentiat	EDF1;EDF1 RCJMI	5	5	5	44.6	44.6	44.6	16.363	0	9.9375	104000	8	0	0	0	0	0	26359	0	0	17463	0
Q5ZMN1	Uncharacterized protein	G3BP1 RCJMB04_1	2	2	2	5.3	5.3	5.3	52.44	0	7.4674	8860.6	2	0	0	0	0	0	0	0	0	0	1466.8
Q5ZMQ2;P6070(	Actin, cytoplasmic 1 (B	ACTB;ACTG1 RCJI	24	2	2	41.3	9.3	9.3	41.792	0	8.2148	156510	13	0	0	0	0	0	0	0	0	13452	0
Q5ZMT0	14-3-3 epsilon (Fragme	YWHAE;YWHAE I	5	3	3	14.5	11.8	11.8	29.174	0	4.4823	28243	3	15782	0	0	0	0	0	0	0	0	0
Q5ZMW1;Q8AY	Aconitate hydratase, mi	ACO2;RCJMB04_1	9	9	9	15	15	15	85.649	0	37.472	295660	18	0	31067	49046	28578	25218	34361	35164	35455	33927	38499

Q6EE30	Eukaryotic translation elongation factor 1		1	1	1	2.3	2.3	2.3	49.731	0	3.5383	17384	2	0	0	0	0	0	0	0	0	0	4450.8
R4GF71;Q6WEE	Thymosin beta	TMSB15B;TMSB4X	4	4	4	68.2	68.2	68.2	5.0526	0	22.218	288620	19	50829	0	17908	0	73945	28510	23214	97295	47548	23001
Q76MS9	Musculoskeletal embry	MUSTN1 PD284	3	3	3	24.4	24.4	24.4	8.6848	0	5.0844	14726	2	0	0	0	0	28610	0	0	0	0	0
Q7LZE8	Fructose-bisphosphate aldolase (EC 4.1.2.13		3	3	3	100	100	100	2.3436	0	165.64	22148000	100	2946000	2505200	1457400	4234100	4E+06	2240100	1060000	1477300	2594200	1220900
Q7SX63;B3VHV	Heat shock 70 kDa prot	hsp70;HSP70;HSP7	25	14	14	39.1	28.4	28.4	69.912	0	59.298	679080	50	66183	69528	67682	37645	33703	75665	61631	119400	93088	88914
Q7ZZK3	Alpha-1,4 glucan phosphorylase (EC 2.4.1.1		8	3	2	6.4	2.9	1.6	98.564	0	7.085	150690	7	0	0	0	0	0	0	0	0	26173	0
Q8AWI4;Q9073	Alpha-tropomyosin;Alp	TPM1	36	2	2	63	3.9	3.9	32.81	0	3.1415	48417	5	0	0	0	0	0	0	15996	0	0	0
Q8JG64	Protein disulfide-isome	PDIA3 ERP57 GRP	9	9	9	20.4	20.4	20.4	56.181	0	25.878	203200	25	29620	16815	20619	23197	25988	0	23909	37206	23461	21952
Q8JHF6	Pyruvate carboxylase (I	PYC	1	1	1	1.4	1.4	1.4	127.14	0.00533	2.8307	3637.7	3	0	0	0	0	0	0	0	0	135.28	0
Q8QFT5;Q9PRL	Acyl-CoA-binding prot	DBI	3	3	3	37.9	37.9	37.9	9.8051	0	6.1269	31259	5	0	0	0	0	0	0	0	9920.7	0	0
Q8QGU2	Peptidylprolyl isomeras	FKBP12.6 FKBP1B	1	1	1	12	12	12	11.957	0	3.8376	8617.8	2	0	0	0	0	5377	0	0	0	0	0
Q90577-2;Q9057	Sarcalumenin	SRL	14	14	14	19	19	19	86.634	0	67.954	494150	39	83370	63408	58034	27291	25290	32211	81381	31306	53358	58898
Q90593;A0A1D5	Endoplasmic reticulum	HSPA5 GRP78	10	8	8	17.5	15.8	15.8	72.018	0	44.686	197070	12	33995	33309	37451	0	0	0	0	49356	36085	0
Q90838;Q90885;	Leucine zipper protein;	CAVIN1	3	3	3	11.1	11.1	11.1	41.311	0	5.9151	249280	7	0	0	0	0	0	19771	22680	0	0	54325
Q90WF0	CgABP260;CgABP260	actin-binding domai	14	14	14	7.8	7.8	7.8	280.49	0	52.48	246550	33	0	34173	44212	33912	40954	48332	17169	27312	84949	0
Q90YA3;A0A1D	6-phosphofructokinase	pfk;PFKP;PFKP RC	25	25	25	31.6	31.6	31.6	83.924	0	154.64	2408500	108	218710	226650	254960	324260	120500	297360	443810	121310	221440	243280
Q90ZG0	Peptidylprolyl isomeras	FKBP12 FKBP1A	2	2	2	21.3	21.3	21.3	11.973	0	9.4687	337250	11	75731	0	34658	61982	64813	0	0	101360	24122	0
Q92007	Aldolase A (Aldolase C	aldolase C	7	7	7	100	100	100	4.3838	0	207.63	38240000	627	4903900	3327600	4034300	4296400	3E+06	4363000	4707100	4530200	4212400	4069700
Q98953	Protein S100-A6 (Calcy	S100A6	1	1	1	18.5	18.5	18.5	10.276	0	9.5537	2815.9	4	0	0	0	0	0	0	0	0	0	739.77
Q9DEG4	Nebulin (Fragment)		23	12	12	30.3	13.5	13.5	104.69	0	47.939	142440	40	9626.2	22500	22920	10335	7830.8	16224	12172	0	12713	17937
Q9DEG5	Nebulin (Fragment)		23	1	0	31	1.2	0	120.77	0	7.4333	151290	9	0	0	0	0	0	0	0	0	0	28578
Q9DEH4	LIM and SH3 protein;N	lasp-2 NEBL;NEBL	94	94	25	42	42	9.7	277.13	0	323.31	8598900	438	732300	842380	939630	1156100	1E+06	911610	686460	961800	745980	955750
Q9I882	Protein kinase C inhibit	chPKCI HINT1Z	7	7	7	57.1	57.1	57.1	13.759	0	162.94	811220	39	41681	40622	62227	150950	93922	72572	68877	125120	134470	82117
Q9I8D6;Q5ZMG	T-complex protein 1 su	RCJMB04_2c6;tcp-	2	2	2	5.6	5.6	5.6	57.736	0.00274	2.9712	8784.8	2	8784.8	0	0	0	0	0	0	0	0	0
Q9I9D1;F6T197	Uncharacterized protein	VDAC2	5	5	5	18.4	18.4	18.4	30.197	0	11.954	158850	10	0	26217	29436	0	0	28286	0	0	17664	0
Q9PTY2	Skeletal myosin heavy chain		117	7	0	44.8	3.7	0	222.94	0	45.244	235540	18	0	25353	18385	33688	43647	0	0	58438	52611	38437
Q9W6H0	Mimecan (Osteoglycin)	OGN	6	6	6	24.5	24.5	24.5	33.179	0	23.183	408630	27	33394	36642	30690	43301	31401	34733	26625	75722	60763	35600
Q9YH14	Progesterone receptor b	rbf ATP5ME RCJM	2	2	2	33.8	33.8	33.8	8.3387	0	4.4289	28182	4	0	14023	0	0	0	0	0	0	0	0
R4GI80	Uncharacterized protein	SYNPO	4	4	4	6	6	6	117.82	0	20.236	29927	20	0	0	0	0	0	0	7177.9	0	12720	7283.8
R4GIG1;P02565	Myosin-1B (Myosin he	MYH1B MYH3;MY	117	3	3	42.3	2.2	2.2	222.81	0	33.458	192730	11	0	0	25606	0	0	31441	40247	0	21996	0
R4GIV3	Uncharacterized protein	CMYA5	3	3	3	1	1	1	498.92	0	6.8531	10039	4	0	0	0	0	0	0	0	0	0	1024.3
R4GKM8	Uncharacterized protein	CAVIN4	4	4	4	11.2	11.2	11.2	40.664	0	9.387	62953	7	0	0	0	0	22831	0	0	0	0	0
R4GL30	Uncharacterized protein	BAG2	2	2	2	9.4	9.4	9.4	25.395	0	4.3195	1497.5	3	0	0	0	0	0	163.96	0	0	0	0
R4GM10	Fructose-bisphosphate	ALDOC	9	9	7	19.5	19.5	17.6	39.36	0	203.19	16305000	223	1581300	1768000	1904700	2022400	1E+06	1711100	1739800	1888500	1931000	1829800
R4GME1	Prefoldin subunit 2		2	2	2	12.4	12.4	12.4	17.616	0	4.4339	7604.2	3	0	0	0	11010	0	0	0	0	0	0
