# Supplementary material for: Revealing the global mechanism related to carnosine synthesis in the pectoralis major of slow-growing Korat chickens using a proteomic approach
Source: Anim Biosci. 2024 Aug 14;37(10):1692–701. doi: 10.5713/ab.24.0119 (PMC11366509; doi:10.5713/ab.24.0119)
Supplement: Supplementary file 5 [file ab-24-0119-Supplementary-Table-5.pdf]

Table S5. The list of proteins exclusively identified in the Low-carnosine group

Protein ID	Protein name	Gene name
A0A1D5P470	Glutathione S-transferase	*GSTT1*
A0A1D5P893	Poly(rc)-binding protein 2 isoform	*PCBP2*
A0A1D5PDV6	Ribosomal protein S19	*RPS19*
A0A1D5PFA5	Myosin, light chain 10, regulatory	*MYL10*
P15989	Collagen alpha-3(VI) chain	*COL6A3*
A0A1D5PMT8	60S acidic ribosomal protein P2	*RPLP2*
A0A1D5PN46	Fructose-bisphosphatase	*FBP2*
A0A1D5PPF8	Heat shock 70kDa protein 4	*HSPA4*
A0A1D5PG30	Elongation factor 1-delta isoform	*EEF1D*
F1NHW5	Cytochrome b-c1 complex subunit 6	*UQCRH*
E1BVU4	SH3 domain-binding glutamic acid-rich-like protein	*SH3BGR*
F1NCA2	Glycerol-3-phosphate dehydrogenase	*GPD2*
F1NY09	Uncharacterized protein	*C1H11ORF54*
P07630	Carbonic anhydrase 2	*CA2*
