# Supplementary material for: Revealing the global mechanism related to carnosine synthesis in the pectoralis major of slow-growing Korat chickens using a proteomic approach
Source: Anim Biosci. 2024 Aug 14;37(10):1692–701. doi: 10.5713/ab.24.0119 (PMC11366509; doi:10.5713/ab.24.0119)
Supplement: Supplementary file 6 [file ab-24-0119-Supplementary-Table-6.pdf]

Table S6. The list of proteins exclusively identified in the High-carnosine group

Protein ID	Protein name	Gene name
P80226	Fatty acid-binding protein, liver	*LBFABP*
A0A1D5NZY9	Myosin, heavy chain 7B	*MYH7B*
P27731	Transthyretin	*TTR*
F1NK96	Protein disulfide isomerase family A, member 6	*PDIA6*
F1NRM8	Endoplasmic reticulum resident protein 29	*ERP29*
Q02960	Macrophage migration inhibitory factor	*MIF*
Q5ZHP2	O-acetyl-adp-ribose deacetylase 1	*OARD1*
Q5ZKE7	UMP-CMP kinase	*CMPK1*
Q5ZMC0	Endothelial differentiation-related factor 1	*EDF1*
Q90838	Polymerase I and transcript release factor	*PTRF*
R4GI80	Synaptopodin	*SYNPO*
