# Supplementary material for: Revealing the global mechanism related to carnosine synthesis in the pectoralis major of slow-growing Korat chickens using a proteomic approach
Source: Anim Biosci. 2024 Aug 14;37(10):1692–701. doi: 10.5713/ab.24.0119 (PMC11366509; doi:10.5713/ab.24.0119)
Supplement: Supplementary file 7 [file ab-24-0119-Supplementary-Fig-1.pdf]

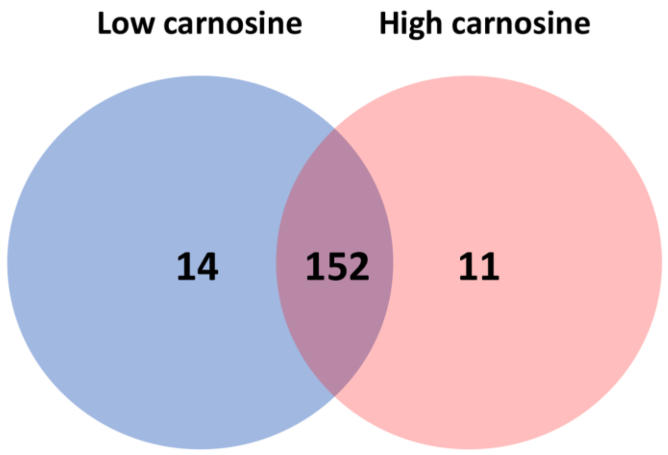

Figure S1. Venn Diagram representing the number of common proteins (center) and exclusive proteins identified in the KRC breast meat in low- (left) and high-carnosine content (right)
